# Synthesis of Protocols for Frostbite Care From the FROST Study Sites

**DOI:** 10.1093/jbcr/irag111

**Published:** 2026-07-01

**Authors:** Rachel M Nygaard, Emily Colonna, Alexander Poole, Lee D Faucher, Rosemary E Paine, Naveen Sangji, Alexandra Lacey, Dhaval Bhavsar, Kristin Colling, Alexis Armour, Irma Fleming, Arek Wiktor, Lucy Wibbenmeyer, FROST multicenter study team, Joshua S Carson, Elisha G Brownson, Damien W Carter, John Loftus, Nicholas Meyer, Kyle Schmitz

**Affiliations:** Department of Surgery, Hennepin County Medical Center, Hennepin Healthcare, Minneapolis, MN 55415, United States; Department of Surgery, Hennepin County Medical Center, Hennepin Healthcare, Minneapolis, MN 55415, United States; Department of Surgery, Whitehorse General Hospital, Whitehorse, Yukon (YT) Y1A 3H7, Canada; Department of Surgery, University of Wisconsin-Madison, Madison, WI 53792, United States; Department of Surgery, MaineHealth Maine Medical Center Portland Surgery, Portland, ME 04102, United States; Department of Surgery, University of Michigan, Ann Arbor, MI 48109, United States; Department of Surgery, Regions Hospital – The Burn Center, Saint Paul, MN 55101, United States; Department of Plastic and Reconstructive Surgery, University of Kansas Health System, Kansas City, KS 66160, United States; Department of Surgery, Essentia Health-St. Mary’s Medical Center, Duluth, MN 55805, United States; Department of Surgery, University of Alberta, Edmonton, T6G 2B7, Canada; Department of Surgery, University of Utah, Salt Lake City, UT 84132, United States; Department of Surgery, University of Colorado, UCHealth Burn and Frostbite Center, Aurora, CO 80045, United States; Department of Surgery, University of Iowa Health Care Medical Center, Iowa City, IA 52242, United States

**Keywords:** frostbite, frostbite management, frostbite treatment protocol, freezing cold injury, frostbite protocol, frostbite clinical management, clinical protocol, clinical management

## Abstract

Frostbite injuries can have lasting sequelae including amputations, intolerance to cold, chronic pain, paresthesia, and inability to return to work. Despite this morbidity, the treatment of frostbite remains understudied. The FRostbite ObServational Trial (FROST) aims to address this gap through a multicenter evaluation of treatment practices and outcomes. As part of FROST, we reviewed institutional frostbite care protocols from 16 participating sites across North America. Practices were categorized as demonstrating uniform agreement (≥90% mentioned the practice), consensus (*≥*75%), or majority if (*≥*50%). Uniform agreement was limited to use of rapid rewarming and thrombolytics, the latter being influenced by site selection criteria for FROST participation. Consensus was achieved in multiple areas (such as urgency of treatment, perfusion assessment, the therapeutic windows for pharmaceutical interventions, and the therapeutic dosing of IV thrombolytics). Protocols varied on grades for thrombolytic treatment initiation, prostacyclin analog use, postthrombolytic anticoagulation treatment and duration, the role of physical or occupational therapy, splinting, and appropriate wound care. This manuscript provides a synthesis of protocols and expert opinions for the acute management of frostbite injury. This synthesized protocol may serve as a tool for centers to develop their own standard protocol to facilitate care for frostbite patients. Additionally, current opportunities for frostbite research and improving care are detailed in the manuscript. Education regarding prevention of injury and the need for emergent treatment is needed for both the public and the medical community.

## INTRODUCTION

Frostbite is an injury with potential consequences that are financially costly and life altering for the survivor.^1–12^ Intervening on the ischemic limb(s) or digit(s) caused by frostbite is an emergency. To prevent poor outcomes, pharmacologic interventions are time sensitive.^5^^,^^6^^,^^13–15^ Many people with frostbite injuries do not identify the severity of the injury early or face other delays in seeking care, which limits access to pharmacologic treatments and results in poor outcomes.^5^^,^^7^^,^^8^^,^^12^^,^^16–23^ Thirty percent or more of those afflicted with severe frostbite will need an amputation.^7^^,^^8^ This is especially concerning, as the majority of civilian patients with frostbite injuries are unhoused, financially impoverished, or suffer from mental illness.^4^^,^^6–8^^,^^11–13^^,^^16^^,^^24^

The pathophysiology of frostbite has been delineated in both basic science and animal models.^17^^,^^25–28^ Frostbite injury involves tissue ischemia, inflammation, coagulation, and reperfusion injury.^17^^,^^28^ As the tissue cools, blood flow becomes sluggish, the tissue becomes ischemic, inflammatory mediators are produced, and coagulation occurs. The damage resulting from this stage may be reversible. However, the degree of injury is additionally impacted by the reperfusion injury. With rapid rewarming, the inflammatory mediator cascade progresses as blood flow to the frozen extremities is restored.^29–37^ This rewarming process results in vascular stasis, inflammatory cell adhesion and migration, edema, and microvascular thrombosis.^17^^,^^28^ Blister fluid from frostbitten hands contains elevated levels of prostaglandins and thromboxanes; however, it is still unknown if the edema is caused by these vasoconstricting, platelet-aggregating, and leukocyte-adherent prostanoids, or if they are a result of white blood cell action in the capillaries.^38–40^ While the rewarming process may reverse the ice crystal formation and vasoconstriction, severe frostbite carries the potential for recurrent stasis, tissue ischemia, and eventual tissue death if timely therapeutic treatments are not provided.^5^^,^^6^^,^^10–13^^,^^24^

The current acute pharmacologic treatment, consisting of thrombolytics and prostacyclin analogs, has successfully reduced amputation rates by up to 80%.^5^^,^^6^^,^^10–13^^,^^24^ Unfortunately, based on single-center reports, approximately half of severe frostbite patients do not receive pharmacologic intervention.^5^^,^^6^^,^^10–13^^,^^15^^,^^24^^,^^41–45^ Most of these cases are due to prolonged exposure with freeze–thaw cycles and delays in obtaining medical care.^12^^,^^21^^,^^23^^,^^24^^,^^42^^,^^44^ Moreover, thrombolytics have a significant list of contraindications which further limit patient access.^23^^,^^27^^,^^46^ Prostacyclin analogs are titrated to patient tolerance, which may also contribute to lower than optimal dosing or reduced duration of treatment.^10^^,^^47^^,^^48^

With climate change leading to intermittent freezing temperatures in more southern areas, up-to-date treatment protocols for frostbite are needed to extend knowledge of its management to other centers.^22^^,^^49^ One of the first comprehensive protocols described regarding frostbite care was published in 1983, and few manuscripts have provided detailed treatment protocols since the addition of thrombolytics and prostacyclin analogs.^10^^,^^12^^,^^21^^,^^41^^,^^50^ As part of the Frostbite Observational Trial (FROST), this article reviews and characterizes frostbite treatment protocols across 16 high-volume frostbite centers in North America. This synthesis of current practices provides a snapshot of contemporary care and may serve as a resource to guide institutions in developing or refining their own protocols. The overarching goal is to foster dialog and collaboration, enhance the quality of frostbite care, and ensure that hospitals have access to expert-informed approaches to management.

## METHODS

Institutional protocols for frostbite treatment were aggregated across 16 centers in North America that routinely treat frostbite patients. Twenty centers expressed interest in participating in the FROST study. Diverse centers were selected based on their use of pharmacologic intervention (thrombolytics and/or prostacyclin analog treatments) and research infrastructure. One selected center withdrew due to institutional requirements for grants administration. That center is included in this manuscript, because this study is not based on patient outcomes, but on clinical protocols at active sites.

Frostbite Observational Trial is a multicenter, prospective, observational study designed to harmonize data collection across 15 North American institutions, with the long-term objective of improving functional limb and digit salvage following severe frostbite injury. Frostbite Observational Trial was developed in response to a critical gap in high-quality evidence for frostbite management. The relative rarity of severe frostbite at any single center and the seasonality of the injury have historically precluded the kind of robust, multicenter data needed to guide clinical protocols. Funded through the FY23 Military Burn Research Program of the Congressionally Directed Medical Research Programs of the Department of Defense, the study has 5 primary aims: evaluating the impact of warm ischemia time, the accuracy of visual assessment relative to formal perfusion imaging, the impact of early pharmacologic intervention (ie, thrombolytics) and rapid rewarming on tissue salvage, and the safety of early thrombolytic delivery prior to formal imaging. The present analysis of treatment protocols represents a characterization of current practice variations at participating centers.

**Figure 1:**
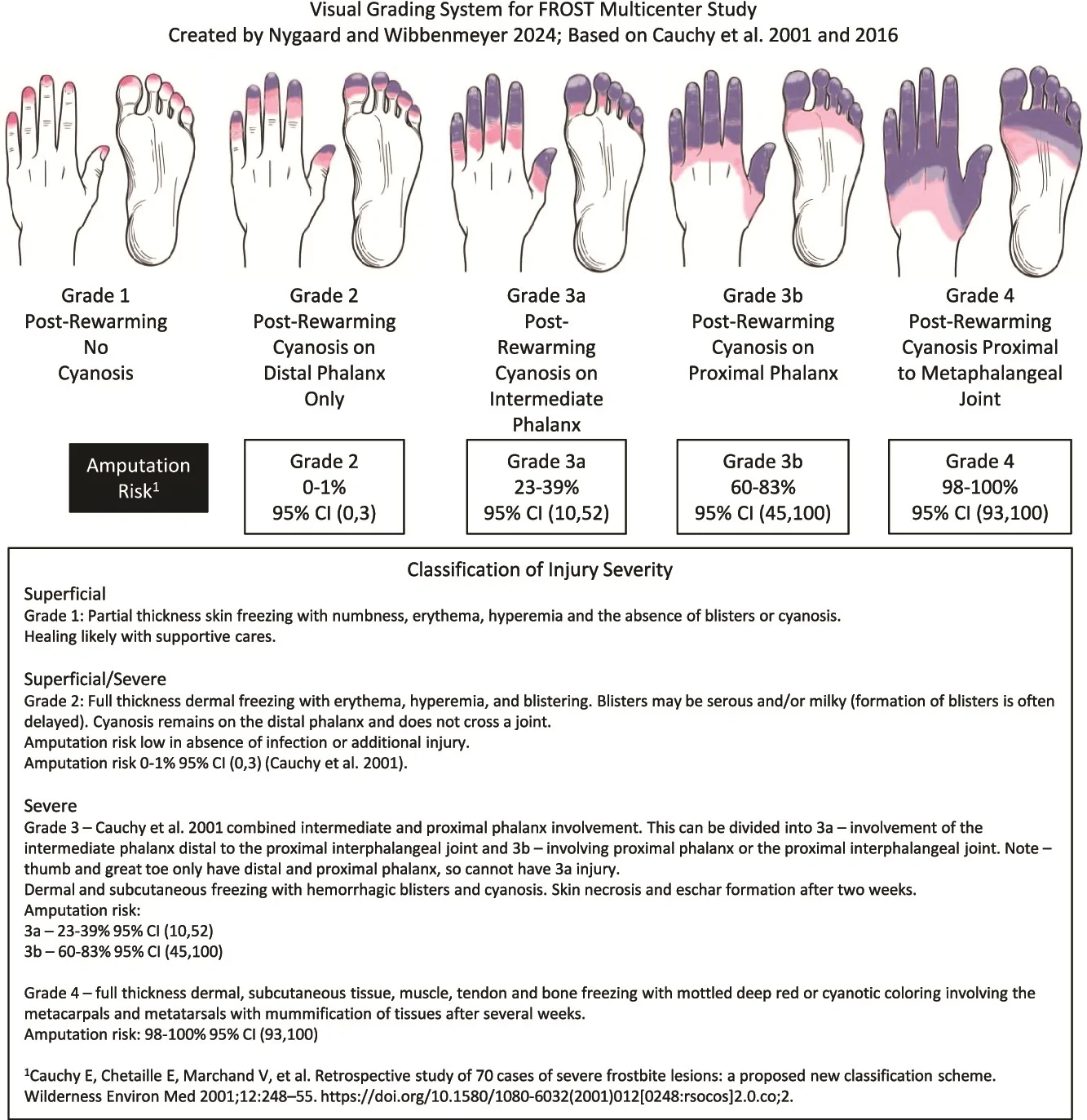
Visual Grading for Assessment of Severity of Frostbite Injury. Each Digit Is Graded Independently and Treatment Is Based on the Most Severe Grade

This article focuses only on atmospheric cooling of tissues and not on contact frostbite, as the pathology, treatment, and outcomes differ. Severe frostbite is defined as a postrewarming perfusion deficit in limbs, digits, and/or tissues. This can be quantified by visual assessment and/or perfusion imaging. The Cauchy visual grade (Figure 1 association with risk of amputation) and/or the Hennepin Frostbite Score (a tool to evaluate outcomes of interventions) are used in the literature to assess the amount of tissue impacted by the injury (both scores) and tissue salvage (Hennepin Score).^51^^,^^52^ While frostbite can impact multiple areas, fingers and toes comprise the majority of the body impacted by frostbite and are the focus of these protocols.

Three reviewers independently assessed the protocols, denoted the presence or absence of specific treatments, and detailed the timing and medical management. Disagreement was moderated by additional review of protocols by reviewers to establish consensus. When a protocol question remained after additional review, the center’s site-principal investigator was contacted to provide additional documentation and clarity. Final tables were created after each center reviewed and approved their accuracy. Summative data were calculated and tables were generated including: overall management (Table 1), rewarming (Table S1), perfusion diagnostics (Table S2), timing and indications for thrombolytic therapy (Table S3), dosing for thrombolytic interventions (Table S4), dosing for prostacyclin analogs (Table S5), contraindications for thrombolytics (Table S6), cautions for thrombolytics (Table S7), contraindications and cautions for prostacyclin analogs (Table S8), posttreatment medications (Table S9), rehabilitation practices (Table S10), and wound and blister care (Table S11). A synthesized protocol was developed based on the compilation of protocols (Figure 9; File S1).

**Table 1:** Overall Management by Center

Management aspect	Percent concurrence	A	B	C	D	E	F	G	H	I	J	K	L	M	N	O	P
Urgent treatment for limb salvage	81	x	x	x	x	x	x	x		x		x	x	x		x	x
Rapid rewarming	94	x	x	x	x	x	x		x	x	x	x	x	x	x	x	x
Pain management																	
NSAIDs (ibuprofen, naproxen)	94	x	x	x	x	x	x	x	x		x	x	x	x	x	x	x
Opioids (morphine, fentanyl)	88	x	x	x	x	x	x		x		x	x	x	x	x	x	x
Gabapentin	69	x	x	x		x	x		x			x	x	x		x	x
Nerve blocks	6	x															
Perfusion imaging before thrombolytics	75		±	±	x	x	x	x		x		x	x	^*^		x	x
Perfusion imaging after thrombolytics	75		x	x	±	x		x	x	x	x	x	x	x		x	
Perfusion assessment																	
Visual	81	x	x	x	x		x	x	x			x	x	x	x	x	x
Doppler	56		x	x	±		x	x			x		x			x	x
ICG angiography (LUNA/SPY)	38				x	x				x			x			x	x
Angiography	44		x				x	x		x	x		x	x			
Tc99 scan	63		x	x		x	x		x	x	x	x		x			x
SPECT	0																
Thrombolytics: IV	88	x	x	x	x	x	x		x	x		x	x	x	x	x	x
Thrombolytics: IA	44		x				x	x		x	x		x	x			
Prehospital thrombolytic administration	44	^**^		x	^***^	^***^	x						x	x			
Prostacyclin analog	63	x	x	x		^	x			x		x	^	x	x	x	x
Postthrombolytics anticoagulation:																	
Enoxaparin/tinzaparin	75	x	x	x	x	x	x		x	x			x	x	x	x	
Heparin	88	x	x	x	x		x	x	x	x	x	x	x	x		x	x
Warfarin	19				x			x	x								
Aspirin (81 mg daily)	75	x		x	x	x	x		x	x		x	x	x		x	x
Topical treatments (antiseptics, hydrotherapy)	94	x	x	x	x	x	x	x	x		x	x	x	x	x	x	x
Delayed amputation consideration	50		x		x	x				x		x	x	x		x	

± Consider; ^*^IA dosing only; ^**^consider starting and transferring; ^***^does not have protocol, but does receive patients with thrombolytics initiated at outside centers; ^has protocol, but not currently implemented.

The article is divided into sections that cover each aspect of frostbite care, from initial treatment to subsequent therapeutic options, wound care, mobilization, and surgical treatment. Each section is followed by opportunities for additional collaboration and research. The data were quantified using the following terms: uniform agreement if *≥* 90% mentioned the practice; consensus if *≥* 75% mentioned practice; and majority if *≥* 50% mentioned the practice. These cutoffs were identified prior to the generation of the synthesis protocol and represent levels of agreement between the protocols at centers with experience managing high volumes of frostbite injuries. Each author additionally contributed to this manuscript and approved the detailed synthesis of protocols. Descriptive statistics were calculated in the tables. Each center has institutional review board approval for participation in the FROST study; however, the data presented in this manuscript do not include human subjects research. The level of evidence reported in this manuscript is level 5: expert consensus.

## RESULTS

An overview of treatment protocols is detailed in Table 1. Each subsection will indicate the tables included in the Supplementary Materials.

### Initial treatment of frostbite (Table S1)

As frostbite often occurs in the setting of hypothermia, attention is first directed toward treating hypothermia, as this is life-threatening. Treatment for hypothermia is detailed in other literature and is outside the scope of this manuscript.^53–55^ Once the core temperature is addressed, attention turns to rewarming the frostbitten extremities. Rapid rewarming in a circulating, warm water bath is the most consistently endorsed initial intervention in the literature, preceding pharmacologic therapies.^31–37^^,^^46^^,^^53^^,^^55^^,^^56^ The therapeutic rationale is rooted in the underlying pathophysiology of frostbite, which involves direct cellular injury from ice crystal formation and secondary injury from a cascade of reperfusion-related mediators resulting in vascular compromise.^38–40^ By minimizing the time tissues remain frozen, rapid rewarming mitigates endothelial damage, microvascular thrombosis, and the inflammatory responses that contribute to irreversible tissue loss. Compared to gradual or spontaneous rewarming, this technique has been shown—particularly in animal models—to significantly reduce necrosis and preserve viable tissue.^34–36^^,^^57^^,^^58^ While relatively simple in concept, rapid rewarming faces challenges, including the need for ingenuity to perform the procedure in the emergency room and a widespread lack of knowledge regarding the process.^29–37^ Importantly, it must also be performed in settings where refreezing is no longer a risk and access to therapeutic treatment is timely, and, as this warming is intensely painful, pain should be managed with analgesics.^31^^,^^55^

Although mentioned in the literature, specific details related to methodology are limited and poorly studied. Rapid rewarming was studied by Mills et al. in the 1960s, who reported improved limb length salvage and tissue preservation with rapid rewarming.^33–36^ Despite this benefit, the practice is not standard across all centers in the current treatment of frostbite.^37^ Rapid rewarming involves immersing the frozen extremity in circulating water at a temperature of 38-42°C for 15-30 min or until the tissue becomes pliable and erythematous. Because rapid rewarming is mechanistically and ergonomically challenging, some have reported using a sous vide to circulate and heat the bath at a constant temperature.^29^^,^^30^^,^^59^ Passive rewarming (forced air-warming systems, warm blankets, and warm intravenous fluids) is less effective and should not be used alone when rapid rewarming is feasible. Regardless of the warming method, the severity of the injury must be assessed postwarming to determine the need for further interventions.

**Figure 2:**
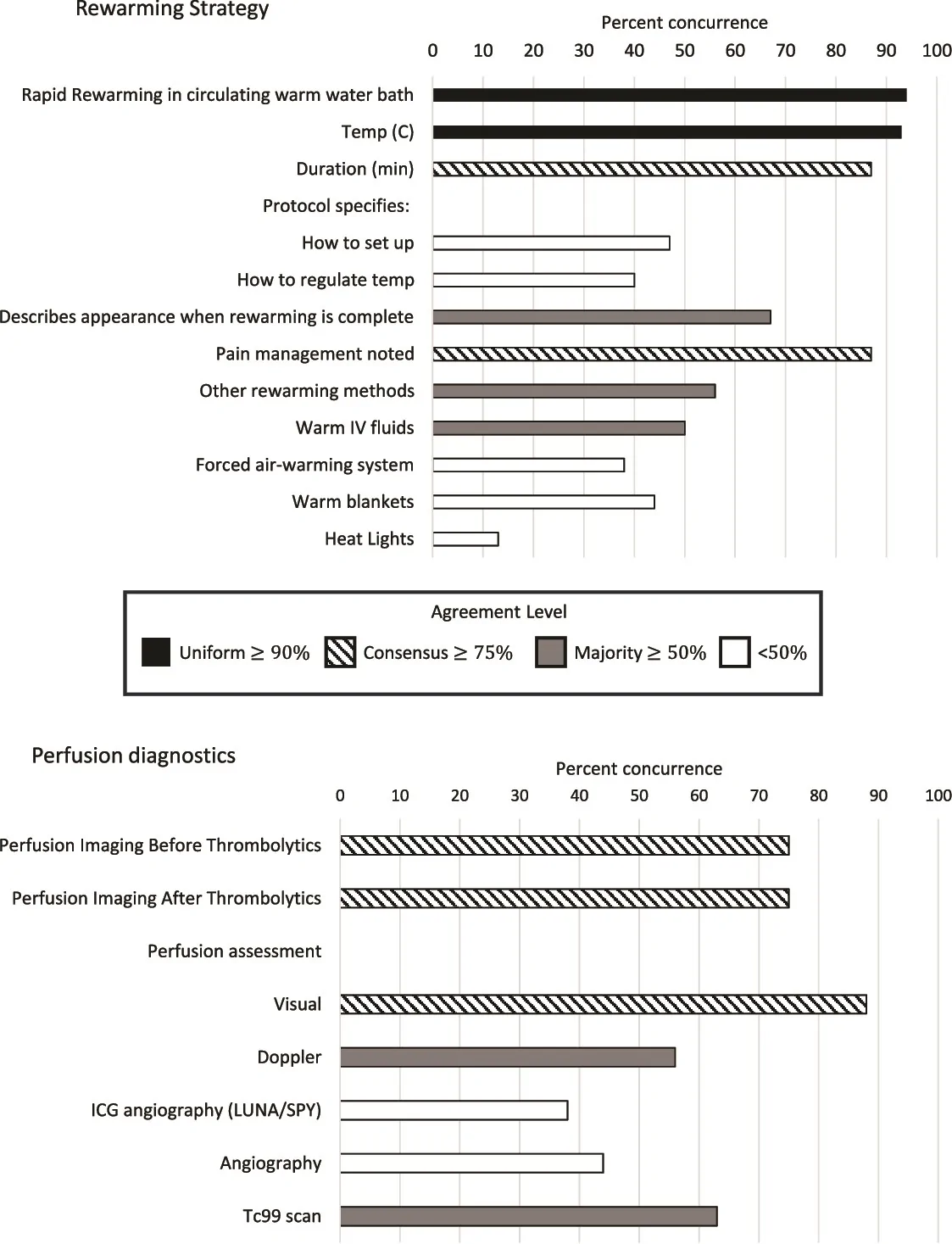
Visual Representation of Protocol Concurrence on Rewarming Strategies (Table S1) and Perfusion Diagnostics (Table S2)

*Protocol review* (Table S1): Use of rapid rewarming (94%) reached uniform agreement. Of those that mentioned rapid rewarming, the need to set a temperature during the performance of the rapid rewarming (93%) reached uniform agreement. The duration for the rapid rewarming (87%) and the need for pain management (87%) reached consensus agreement. Other rapid rewarming process details, including how to set up the water bath and how to maintain the temperature during rewarming, were more sparsely mentioned in the protocols (Figure 2).

***Opportunities:*** One aim of the FROST multicenter study is to evaluate the impact of rapid rewarming in a large cohort of patients across North America. While initial treatment of frostbite may occur prior to definitive hospital admission, centers need to have detailed protocols for the rapid rewarming process, including setup, target temperature, temperature maintenance, and duration. Pain management also needs to be uniformly addressed in all protocols. Questions remain related to the timing of the initiation of pharmacological intervention and the completion of rapid rewarming. In the setting of a clear, severe grade 3 or 4 injury, clinical practice guidelines support the initiation of other interventions prior to the completion of postrewarming perfusion imaging, particularly when rewarming is delayed or slow.^31^^,^^55^ While relatively rare, some patients arrive with a significant grade 4 injury proximal to the carpals and/or tarsals. Warming this injury may be slow, so the timing of pharmacologic intervention (in the absence of a requirement for extracorporeal membrane oxygenation) prior to the completion of rewarming should be deliberated among providers during protocol development. Additional research and widespread education for providers may help facilitate this cumbersome process and enable more widespread adoption. Innovation is needed for safe and effective methods to facilitate rewarming in both hospital and remote field settings.

### Diagnostics for assessment of frostbite (Table S2)

Advanced therapeutic treatment depends on the timely recognition and diagnosis of severe frostbite. Diagnosis based on clinical signs includes numbness, pallor, and motor impairment in the affected extremities.^17^^,^^51^^,^^55^^,^^60–62^ However, the initial clinical appearance can underestimate the depth of injury, especially before thawing.^63^^,^^64^ Prognostic evaluation should occur postthaw; while imaging modalities are often used to help delineate the extent of perfusion deficits, the initiation of treatment should not be delayed to obtain imaging in the setting of clear, severe frostbite.^31^^,^^55^ Historically, in the United States, frostbite has been diagnosed and classified similar to burns, based on the depth of the injury, with first-(frostnip), second-, third-, and fourth-degree injuries. Alternatively, it was more simply classified as superficial (absence of a postrewarming perfusion deficit) and severe or deep (presence of a postrewarming perfusion deficit).^17^^,^^28^ The prognostic visual grading scale was introduced by Cauchy et al. and is not equivalent to the degree or severity of the injury, but rather indicates the risk of amputation based on the amount of tissue impacted by severe injury.^51^^,^^65^ The system classifies injuries based on the level of cyanosis after rewarming, with grade 1 (no cyanosis) being a superficial injury and grades 2-4 representing progressively more proximal, severe injury (Figure 1).^62^ One major concern with visual assessment alone is that ischemia is challenging to assess visually, particularly in patients with darker skin tones or for clinicians with limited exposure to frostbite injuries.^66^ In the United States, perfusion imaging has classically been the method to detect severity. Initially, Twomey and colleagues used technetium (Tc-99) bone scans, with those lacking perfusion on imaging receiving thrombolytics.^67^ More recently, practices have shifted away from formal imaging toward either ICG fluorescence angiography or to no imaging prior to treatment.^13^^,^^31^^,^^68–71^

*Protocol review* (Table S2)*:* There was no uniform use of perfusion assessments—with the majority using some type of assessment prior to and after thrombolytic therapy. Overall, 75% of centers still use some method of perfusion assessment (Tc-99 scan, indocyanine green [ICG] angiography, or angiography) to assess eligibility for thrombolysis. Similarly, 75% of centers use perfusion assessment to evaluate posttreatment revascularization. Only 2 centers use visual assessment alone as the means to evaluate the severity of the injury and identify those requiring further pharmacologic intervention. More than half of the centers report use of Doppler, 38% use ICG angiography, 44% use formal angiography, and 63% use Tc-99 scans. Most centers use a combination of imaging modalities, with centers that use intravenous (IV) thrombolytics leaning more toward ICG angiography and/or Tc-99 scans and those that use intra-arterial (IA) thrombolytics using formal angiography (Figure 2).

***Opportunities:*** Studies are needed to compare methods for the initial evaluation of the severity of frostbite. One aim of the FROST multicenter study is to compare visual assessment to more formal methods of perfusion. Worldwide, the Cauchy grading scale is used, but visual assessment can be challenging, particularly when providers are not very familiar with frostbite injuries. Even at high-volume centers, providers understand that an injury may progress over time, and a grade 2 injury may shift to a grade 3 injury after 24 h. To account for this, centers treat grade 2 injuries as they do grade 3, but reassess the injury and treatment as it progresses. Protocols are mixed regarding intervening for grade 2 injuries; however, centers that rely on perfusion imaging would intervene for grades 2-4, given that they all have some remaining postrewarming ischemia.

Any diagnostic methods need to be readily available as frostbite is a time-sensitive injury.^5^^,^^6^^,^^13–15^^,^^47^ The role of perfusion imaging in evaluating revascularization with prostacyclin analog therapy is an area in need of study. This should include an assessment of the dose and duration of therapy compared to imaging and final tissue salvage.

Significant partnerships are needed to develop lower-technology/lower-resource methods of identifying tissue ischemia. Indocyanine green angiography offers the ease of rapid, bedside evaluation of perfusion and surveillance of treatment efficacy.^68–71^ However, more research is needed to determine its role and capabilities. Widespread education and educational tools are needed to train providers in the identification of tissue ischemia, particularly in the setting of varying skin tones. The role of Doppler in the evaluation of perfusion in frostbitten areas is an opportunity needing further study. Combining serial visual assessments, digital pulses, and imaging to delineate progress and the need for further treatment remains a priority.

### Pharmacologic treatment of frostbite (Tables S3-S5)

Urgent pharmaceutical treatment options for grades 2-4 frostbite include thrombolytic therapy (medication used to dissolve blood clots), prostacyclin analog therapy (medication to dilate blood vessels and inhibit platelet aggregation), and their combination, but the comparative effectiveness of these interventions is unclear. The literature supports pharmacologic treatment (thrombolytics and prostacyclin analogs) in those who qualify with grades 2-4 frostbite.^2^^,^^31^^,^^46^^,^^55^^,^^72^^,^^73^

#### Thrombolytics (Tables S3 and S4)

Twomey and colleagues investigated IA thrombolytics to treat severe frostbite within 48 h of rewarming.^67^ They noted improvement of frostbite in untreated limbs, leading to the development of a dosing schema and use of IV thrombolytics for severe frostbite. Although the development of the dosing strategy was never published, its use remains unchanged since the original study.^67^ Since this sentinel study, outcomes following thrombolytics have been reported in over 400 patients with a salvage rate of over 75% (Table 2, calculated weighted average 76.2%). The route of delivery, either IV or IA, does not appear to make a difference (Table 2).^2^^,^^73^ However, this may be more a factor of a reduced warm ischemia time in the setting of IV delivery, as it is faster to initiate and can be started at outside centers prior to transfer.^13^^,^^31^ The most widely studied thrombolytic for frostbite is alteplase. A few centers use the thrombolytic tenecteplase which has a longer half-life, shorter infusion duration, and potential for repeat dosing.^11^^,^^74^^,^^75^ Streptokinase and urokinase have been used historically in the literature and are still used in China.^18^^,^^51^^,^^76^

**Table 2:** Literature Summary of Frostbite Management

Author	Year	Total number of patients	Number of patients treated	Treatment	Imaging	Treated individuals with amputations	Salvage rate for treated individuals	Individuals with amputation after supportive cares	Salvage rate for individuals with supportive cares
Nygaard^5^	2025	131	131	IVTT	Tc99 and ICG angiography	28.2	83.9	–	–
McCormick^77^	2025	5	5	IATT and IVTT	ICG angiography and angiography	20	Unknown	–	–
Carmichael^13^	2021	199	72	IVTT	Visual/Tc99	31.9	68.1	60.6	Unknown
Paine^11^	2020	17	4	IATT	Angiography	75	83.3	9.1^*^	Unknown
Heard^12^	2020	99	7	IATT	Angiography	28.6	82	35.7	Unknown
Heard^12^	2020	3	2	IVTT	Angiography	0	100	35.7	Unknown
Al Yafi^45^	2019	18	9	IATT	Angiography	55.6	62.04	66.7	62.82
Bruen^24^	2007	32	6	IATT	Angiography	Unknown	90	Unknown	59
Gonzaga^42^	2016	69	62	IATT	Angiography	32.3	68.6	Unknown	Unknown
Linford^21^	2017	14	9	IATT	Angiography	66.6	81.1	Unknown	Unknown
Taveri^43^	2016	13	13	IATT	Angiography	30.8	83	–	–
Wexler^78^	2017	6	6	IVTT	None	Unknown	75.4	–	–
Johnson^79^	2011	11	11	IVTT	Tc99	54.5	60.9	-	–
Jones^80^	2017	12	7	IVTT	Tc99	28.6	72.5	Unknown	Unknown
Nygaard^6^	2017	73	45	IVTT	Tc99	36	74	50	55
Twomey^67^	2005	35	19	IATT and IVTT	Tc99	15.8	81	37.5	Unknown
Magnan^81^	2021	58	58	IVPA	Tc99	Unknown	89.9	–	–
Cauchy^15^	2011	47	16	IVPA	Tc99	0	100	60	60.4
Cauchy^15^	2011	47	16	IVTT + IVPA	Tc99	Unknown	96.9	60	60.4
O’Dochartaigh^47^^***^	2026	257	80	IVTT + IVPA	None	32.5	74.6	20.7	79.2
Crooks^48^^***^	2022	90	26	IVTT + IVPA	Tc99	31	74.4	29.5	77.2
Poole^10^^***^	2021	22	22	IVTT + IVPA	None	Unknown	79.6	–	–
Patel^44^^***^	2017	17	8	IATT + IVPA	Angiography	Unknown	85	Unknown	23

Abbreviations: IATT, intra-arterial thrombolytic therapy; ICG, indocyanine green; IVPA, intravenous prostacyclin analog; IVTT, intravenous thrombolytic therapy; Tc99, technetium-99m scan

^*^All very minor injury—details not reported.

^**^Includes 1 patient with thrombolytics delivered > 48 h.

^***^Some patients received both iloprost and thrombolytics—lacking details to delineate combination.

Thrombolytic administration has a narrow therapeutic window after rewarming for successful limb salvage. While the literature cites a therapeutic window of 24-48 h, Nygaard et al. showed that every hour of delay to thrombolytics resulted in a decrease in tissue salvage by 28%, encouraging more expeditious treatment whenever possible.^10^^,^^11^ In a letter to the editor, the only randomized control trial by Cauchy et al. also showed more favorable results when intervention occurred less than 12 h from rewarming.^15^ This was supported by Carmichael et al. demonstrating earlier remote delivery of thrombolytics resulted in lower rates of amputation, though they did not report details of warm ischemia time.^13^ In a more recent study, Nygaard et al. demonstrated benefit to treating with thrombolytics at later timepoints.^5^ This supports the idea that strict treatment windows should not be used, as some patients had full response to thrombolytics even at 15 h postwarming, but urgent treatment with thrombolytics remains a priority.^5^

**Figure 3:**
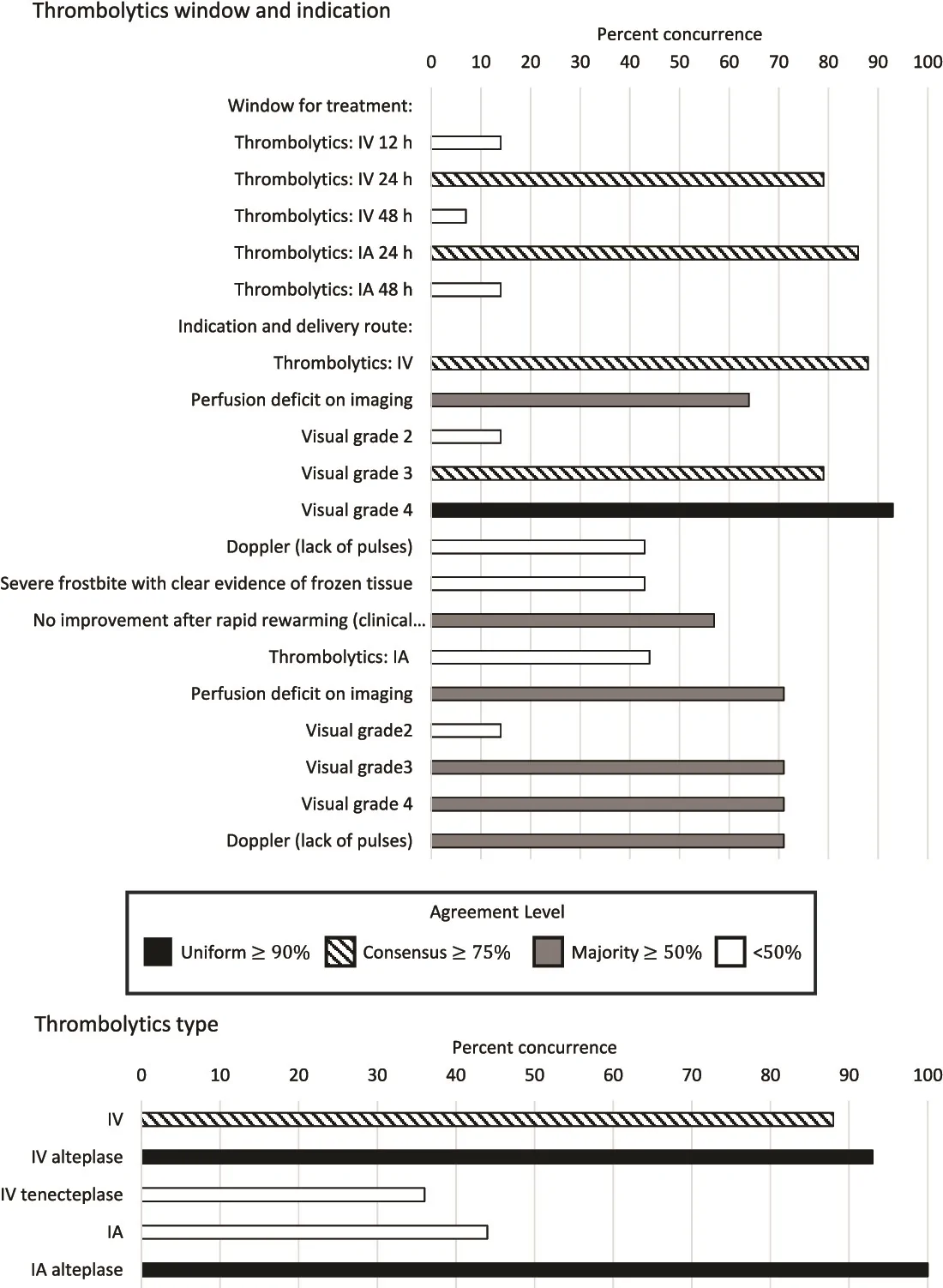
Visual Representation of Protocol Concurrence on Indications for Thrombolytic Therapy (Table S3) and Thrombolytic Medication Dosage and Route of Delivery (Table S4)

*Protocol review for thrombolytic administration* (Tables S3 and S4): There was uniform agreement among the centers for the following: the use of either IV or IA thrombolytics in severe frostbite injury (100%; this was a requirement for participation in the FROST study). Consensus agreement was met for the therapeutic window for thrombolytic treatment within 24 h (both IV and IA). All but 2 centers administer IV thrombolytics, with 7 other centers offering both routes of administration. Centers vary on how to determine when to administer both IV and IA thrombolytics (Table S3). Most administer for both grade 3 or grade 4 injury based on visual assessment and/or lack of perfusion based on imaging to qualify for intervention (this would include grade 2 injury). The majority of centers use alteplase with one center using exclusively tenecteplase and 4 additional centers using tenecteplase in addition to alteplase (Table S4). Authors included in this study report plans to switch to tenecteplase dosing in the coming season. This is due to a mix of availability differences between alteplase and tenecteplase, shorter duration of tenecteplase treatment, and potential for repeat dosing of tenecteplase (personal communications). Dosing details are provided in Table S4 (Figure 3).

#### Prostacyclin analogs (Table S5)

Numerous case reports, larger case studies, and a single randomized trial report outcomes following use of prostacyclin analogs.^10^^,^^14^^,^^15^^,^^18^^,^^44^^,^^47^^,^^48^^,^^71^^,^^76–86^ Recent reviews demonstrated difficulty evaluating the literature due to the low numbers of patients treated and high variability in protocols.^2^^,^^9^^,^^46^ The existing literature comparing prostacyclin therapy in frostbite patients outside of the United States consists of mostly winter sport athletes; this contrasts to the high proportion of persons experiencing homelessness, drug and alcohol abuse, and/or mental health conditions in US frostbite patients.^9^^,^^12^^,^^16^^,^^87–90^

Prostacyclin analogs such as iloprost and epoprostenol were initially developed for treatment of severe pulmonary arterial hypertension (PAH) due to their vasodilatory and antiplatelet effects. Iloprost was recently approved by the US Food and Drug Administration (FDA) for treatment of severe frostbite in the United States; however, it has been used in Europe and Canada for severe frostbite for many years.^9^^,^^10^^,^^15^^,^^47^^,^^48^^,^^82^ Studies evaluating iloprost treatment have demonstrated a decrease in amputation rates relative to untreated patients.^10^^,^^15^^,^^47^^,^^48^ A study by O’Dochartaigh et al. recently reported a dose response for iloprost.^47^ They found a higher total dose (mcg/kg) was associated with a significantly lower likelihood of amputation.^47^ This will likely significantly impact the development of protocols as they are implemented at US centers. In single-center studies, iloprost had a reported success rate of over 80%. This benefit appears to be mostly found in grade 3 injury, as no significant difference in outcomes are reported in grade 2 injury and only one manuscript identified a difference in grade 4 injury (Table 2).^10^^,^^15^^,^^47^^,^^48^ The study by O’Dochartaigh et al. also compared those treated with either iloprost (some were also treated with thrombolytics) and supportive care, they demonstrated amputation in those treated with iloprost or iloprost with thrombolytics resulting in an amputation in 26 out of 80 patients (32.5%) compared to 54 out of 177 (30.5%) supportive care.^47^ They further delineated amputation outcomes by grade of initial injury, finding a statistically significant difference between groups for grade 3 injury (9 out of 30 [30%] iloprost or iloprost with thrombolytics vs 34 out of 65 [52.3%] supportive care, *P* = .042). Another Canadian study by Crooks et al. described digit salvage rates and found significant improvements in grades 3 and 4 injury with iloprost treatment.^48^ An additional complicating factor evaluating this literature is the potential additive effect of thrombolytics used with iloprost for grade 3 and grade 4 injury.^10^^,^^15^^,^^47^^,^^48^

Epoprostenol was the first prostacyclin analog to be approved for PAH treatment nearly 30 years ago and exhibits potent endogenous inhibition of platelet aggregation and vasodilation.^91^ It differs pharmacokinetically from iloprost by exhibiting non-organ-dependent elimination and a shorter half-life (<6 min). Epoprostenol has been well studied in a wide variety of populations exhibiting tissue hypoperfusion such as Raynaud’s phenomenon and digital ischemia/ulcers secondary to scleroderma, CREST syndrome (calcinosis cutis, the Raynaud phenomenon, esophageal dysfunction, sclerodactyly, and telangiectasia), and systemic sclerosis, with a well-established therapeutic index and safety profile.^92–96^

Only 3 patients have been published as individual case reports supporting IV epoprostenol in the treatment frostbite. Bello et al. reported substantial improvement of grade 3 frostbite injury with IA alteplase, heparin, and epoprostenol for 6 days (dose 0.5-2 ng/kg/min).^84^ Due to trauma resulting in a contraindication to alteplase use, Eslami et al. outlined notable improvement of bilateral hand grade 3 frostbite treated with epoprostenol alone.^85^ In contrast, Khan et al. described the use of delayed epoprostenol therapy initiated on the fourth hospital day for grade 3 injury, continued for 5 days, without improvement (maximum dose 6 ng/kg/min).^86^ An ongoing randomized clinical trial evaluating epoprostenol will provide more guidance.^97^ Finally, there is some reported use of alprostadil (prostaglandin E1 analog), but the studies lack detail for a comparison of outcomes.^18^^,^^44^^,^^76^

Although prostacyclin analogs have a longer postrewarming window of delivery, more successful treatment seems likely if, like thrombolytics, it is given urgently following rewarming. A few studies suggest earlier administration of iloprost has superior outcomes.^14^^,^^15^ To date, there are no large studies evaluating the impact of time to treatment using prostacyclin analog therapy, though authors report treating more urgently following the 2017 manuscript by Nygaard et al. and recently reported experience by Dion et al. delivering remote iloprost.^6^^,^^14^

**Figure 4:**
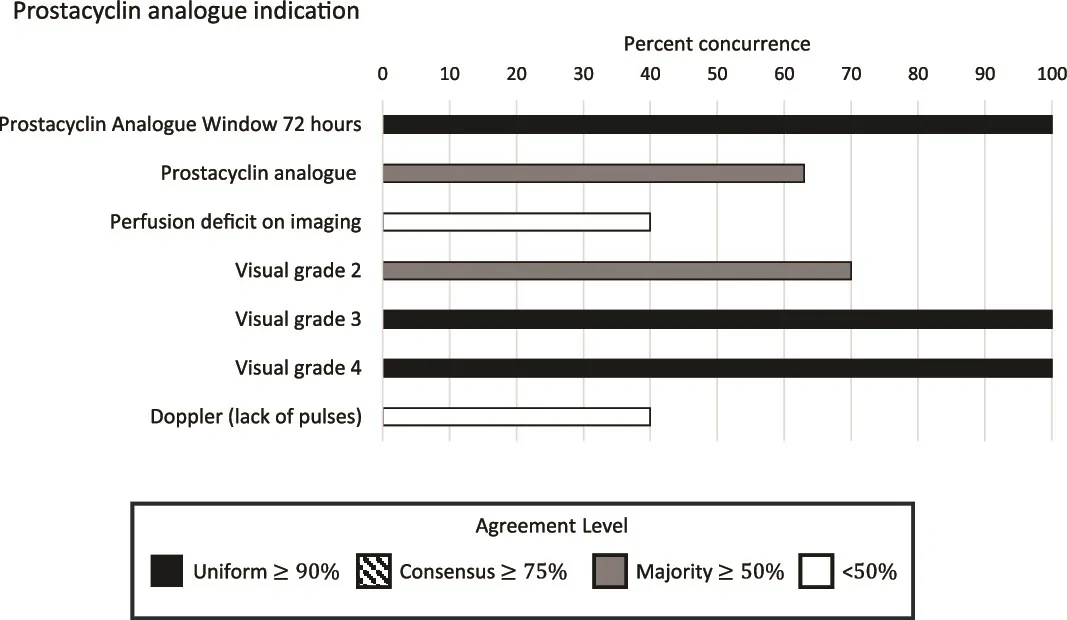
Visual Representation of Protocol Concurrence on Indications for Prostacyclin Analog Therapy and Dosage (Table S5)

*Protocol review for iloprost administration* (Table S5): Of the 10 centers with active iloprost protocols, there was uniform agreement (100%) with the therapeutic window for administering iloprost up to 72 h of warm ischemia time (Table S5). The majority administer prostacyclin analogs for grades 2-4 injury (60%) (Table S5). Most support concurrent delivery of prostacyclin and thrombolytics. Iloprost duration of therapy is typically 6-h infusion for 5 days. The Canadian centers involved in the study by O’Dochartaigh et al. plan to shift toward a total iloprost dosage over the current time-based infusion, but this has yet to be protocolized (personal communication). Duration of epoprostenol therapy is under investigation (Figure 4).

***Opportunities:*** One aim of the FROST multicenter study is to evaluate the impact of pharmacologic interventions on amputation rates and salvage of limbs and digits. Standardizing the clinical indications for delivery of advanced therapeutic care would help advance the evaluation of these methods. While IA and IV routes for delivery of thrombolytics appear equally efficacious, the current literature lacks sufficient detail to determine if either route produces better outcomes based on patterns of injury or treatment windows. At least one center reserves IA delivery for isolated upper extremity injury. Modalities to assess perfusion deficit will be institution specific and use of visual assessment can be challenging for providers less exposed to the injury. Another area of needed clarity is the grade or severity of frostbite that receives pharmacologic interventions. The use of thrombolytics for both grade 3 and grade 4 injuries has strong support in the literature, while many centers also treat grade 2 injury with thrombolytics with documented perfusion deficit after assessment with formal perfusion imaging.^15^^,^^27^^,^^31^^,^^55^^,^^72–75^ Additionally, all centers treat grades 3 and 4 injury and most treat grade 2 injury with prostacyclin analogs, with some leaving grade 2 treatment up to the individual provider or discontinuing after clear delineation of only grade 2 injury later in the treatment course.

While a uniform treatment strategy for delivery would be helpful for a meta-analysis, practices are unlikely to change barring significant demonstration of better outcomes. The impact and utilization of repeat dosing, while done at some centers, is not well described and in need of future research. As frostbite is time sensitive, half of centers have protocols for remote delivery of thrombolytics, but reports are very limited in the literature. Remote delivery of thrombolytics is routine for stroke care and should become routine for frostbite patients where transfer times may result in presentation outside most treatment windows. However, standard protocols to evaluate for potential contraindications are needed to safely implement remote treatment. Additionally, it will be important to carefully evaluate the efficacy of tenecteplase, as there are multiple potential benefits, including a shorter duration of treatment and a longer half-life. The data on iloprost are sparse, as it was only recently approved in the United States. There is an opportunity to determine the optimal treatment window and dosing duration for iloprost. Concurrent rather than staggered delivery of thrombolytics and prostacyclin analogs should be explored. The efficacy of epoprostenol in the management of severe frostbite needs to be evaluated. Additional evaluation of timing and delivery of pharmacologic interventions and methods to safely reduce the impact of warm ischemia time remain a priority.

Finally, opportunities exist to compare the efficacy of prostacyclin analogs in US frostbite populations as well as formal comparison of outcomes after combination therapy with prostacyclin analogs and thrombolytics. There have been no head-to-head comparisons of thrombolytics, prostacyclin analog, or combination therapy with both thrombolytics and prostacyclin analog done in a US population. The studies including thrombolytics and iloprost generally lack detail enough to delineate any specific impact of the monotherapy versus combination therapy. Additionally, most studies outside of the US forego pretreatment perfusion assessment, which makes accurate evaluation of the improvement in perfusion following treatment particularly challenging.

### Contraindications and cautions for pharmaceutical interventions (Tables S6-S8)

#### Contraindications and cautions for thrombolytics (Tables S6 and S7)

Administration of thrombolytics in appropriately screened patients for the treatment of severe frostbite has a low complication rate of 3%-4% that is independent of the route of administration.^72^^,^^73^^,^^98^^,^^99^ Thrombolytics, however, have several restrictions, and only about half of those in the United States with severe frostbite receive thrombolytics.^12^^,^^21^^,^^23^^,^^24^^,^^42^^,^^44^ The reasons for the low administration rate are unclear. While some patients have conditions that are contraindications, many others fall out of the therapeutic window. The reasons for lack of timely care are also largely unknown but include failure to seek medical care secondary to incapacity or inaccessibility. Additionally, there remains general lack of awareness among medical providers and the public that frostbite is an emergency with a narrow therapeutic window for treatment.

Most contraindications to thrombolytic administration are associated with the medication’s complication risk as listed on package labeling. Two contraindications for thrombolytics unique to frostbite treatment are warm ischemia time and freeze–thaw cycles. Neither is associated with an increased risk of complications, but both may reduce treatment efficacy. Providers must balance the risk of complication and risk of loss of digits and/or limbs.^2^^,^^5^^,^^31^^,^^73^^,^^99^ We would argue that saving limbs and digits is an emergency and in the place of patient incapacity to make decisions, every center has protocols for providers to make decisions in the best interest of the patient.^100^

**Figure 5:**
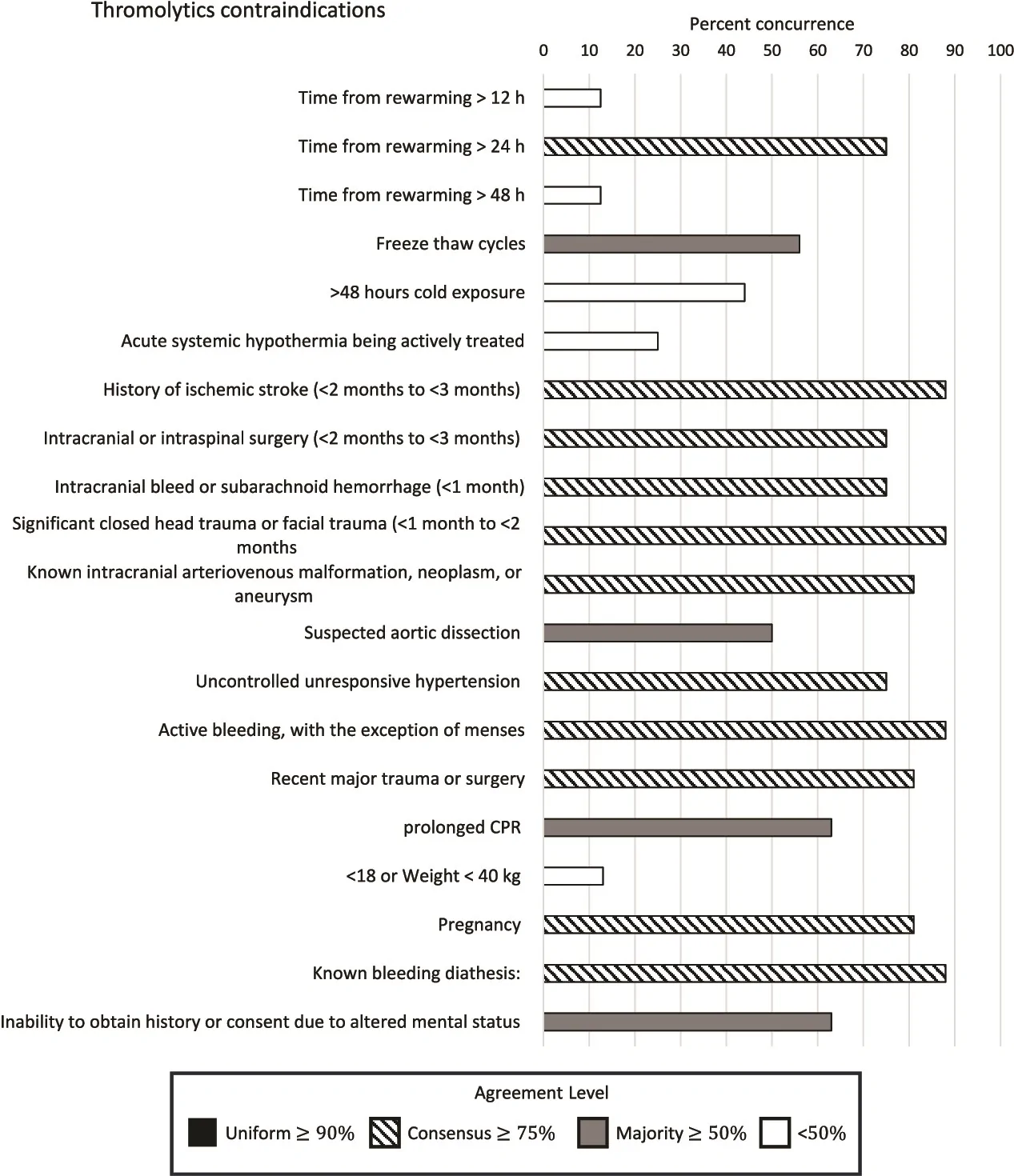
Visual Representation of Protocol Concurrence for Thrombolytics Contraindications (Table S6)

*Protocol review* (Tables S6 and S7): Of the 18 contraindications included in the protocols, there was no uniform agreement. However, there was consensus on over half the contraindications (Table S6). These included: warm ischemia time (75% >24 h), recent ischemic stroke (88%), history of intracranial or intraspinal surgery (75%), recent intracranial bleed or subarachnoid hemorrhage (75%), recent major head or facial trauma (88%), known intracranial arteriovenous malformation, neoplasm, or aneurysm (81%), uncontrolled hypertension (75%), the presence of active bleeding (88%), recent major trauma or surgery (81%), pregnancy (81%), and known bleeding diathesis (88%). Four contraindications reached majority including: freeze thaw cycles (56%), suspected aortic dissection (50%), prolonged cardiopulmonary resuscitation (CPR) (63%), and inability to obtain history or consent (63%). Three contraindications were included in less than half the protocols (Table S6; Figure 5).

**Figure 6:**
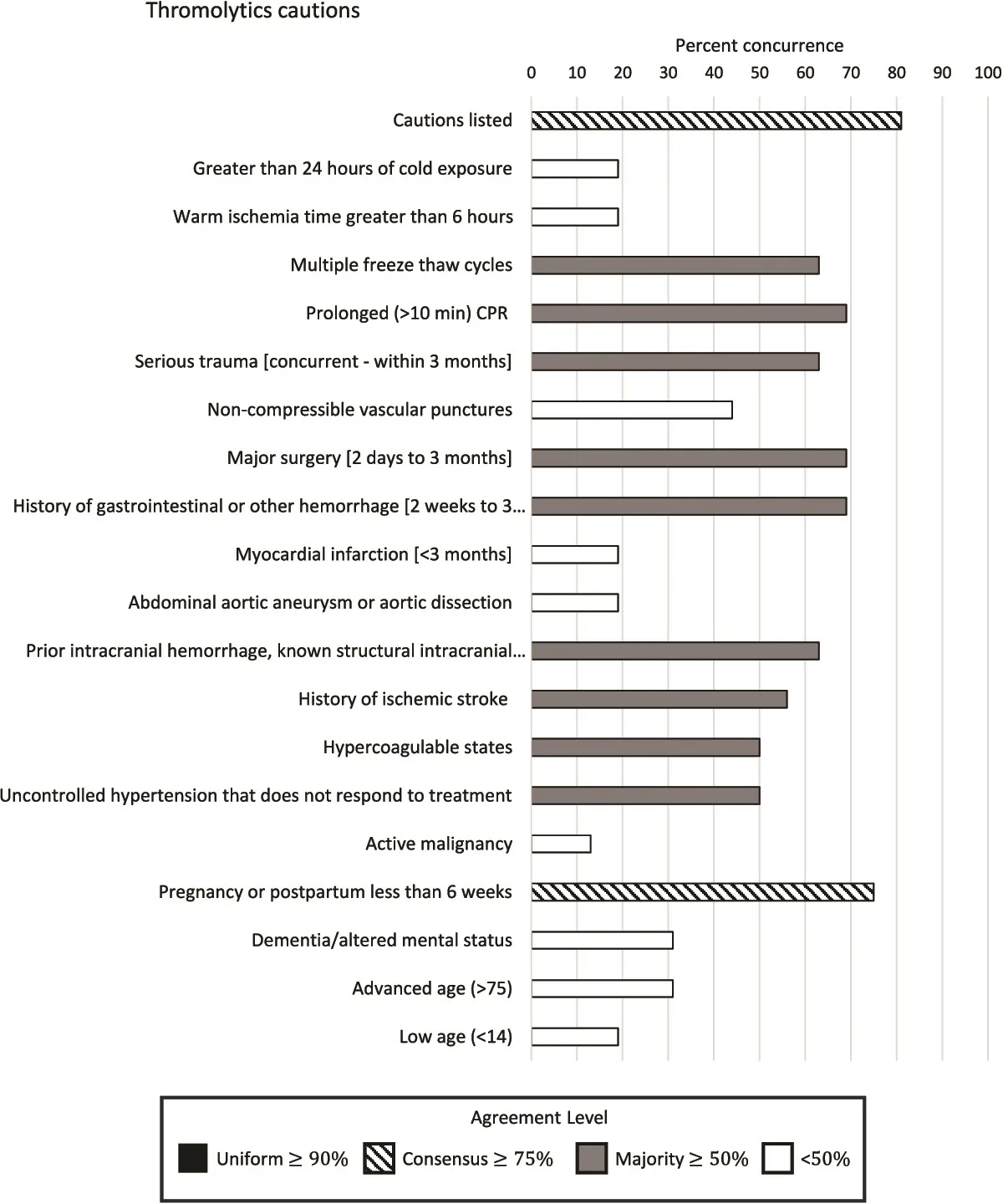
Visual Representation of Protocol Concurrence for Thrombolytics Cautions (Table S7)

Protocols also specify factors that could potentially increase the risk–benefit ratio of thrombolytic administration for frostbite treatment. While not contraindications, these are considered cautions that necessitate informed shared decision-making regarding proceeding with treatment (Table S7). There were 19 cautions mentioned in the 16 protocols. Some centers listed items as both contraindications and cautions to thrombolytic treatment. However, the verbiage was often different making comparisons difficult to draw. No caution was uniformly mentioned. The only caution that reached consensus was pregnancy and being within 6 weeks postpartum (75%). Over one half of the protocols mentioned the following as cautions to thrombolytic treatment: multiple freeze thaw cycles (63%), prolonged CPR (69%), serious trauma (63%), major surgery (69%), history of gastrointestinal or other hemorrhage (69%), history of an intracranial bleed or the presence of intracranial neoplasm or structural lesion (63%), history of stroke (56%), hypercoagulable states (50%), and uncontrolled (uncorrectable) hypertension (50%) (Figure 6).

***Opportunities:*** Harmonization of thrombolytic guidelines is needed to increase the administration of thrombolytics in severe frostbite. There have been similar papers calling for harmonization of thrombolytics in the stroke literature.^100^ Many contraindications have been called into question as the evidence regarding the safety of treatment mounts. There are opportunities to standardize and clarify the language and shift some of the contraindications to cautions. A synthesis of the literature for use of intravascular thrombolytics for ischemic stroke clarifies the contraindications of recent surgery or trauma to within 14 days, instead of within 3 months listed in multiple frostbite protocols.^100^ The same literature shifts history of ischemic stroke, age, dementia, history of myocardial infarction, gastrointestinal bleeding (that is nonstructural), and pregnancy from contraindication to caution. Cautions imply weighing the benefits and the risks so the patient can make an informed decision. Freeze–thaw cycles are sometimes hard to determine and should be moved to a caution. In appropriately screened patients, the complication rate following thrombolytics is low (between 4% and 6%).^73^^,^^99^ Additionally, rather than exclude patients with severe frostbite who present with altered mental status, evaluating their bleeding tendency with laboratory and radiography would limit the risk of therapeutic treatment. Inability to consent should not be a contraindication for thrombolytics as treatment for frostbite is a medical emergency. In medical emergencies, physicians are generally protected by both legal and ethical doctrines to act in the patient’s best interest. The elderly are especially vulnerable to be excluded secondary to age, medical treatment with anticoagulation and antiplatelet drugs, and dementia. Appropriate work-up should be performed to limit their risk as well. Safely adjusting the contraindications and cautions will hopefully reduce these as a barrier for access.

Overall, the frostbite protocols are overly restrictive in terms of conditions that are listed as contraindications (such as history of ischemic stroke, myocardial infarction, and pregnancy), intervals between contraindications and treatment (months rather than weeks), and they lack information on appropriate monitoring during therapy or work-up to allow treatment (treatment to lower blood pressure to acceptable limits or verification of bleeding diathesis). Providers may also benefit from knowing the legality in their practice area regarding emergency treatment. When possible, a legal authorized representative should be approached, however, in emergent procedures in eligible patients where delay will be harmful, it is generally supported by legal and ethical doctrines to proceed without informed consent.

#### Contraindications and cautions for prostacyclin analog therapy (Table S8)

The labeling of iloprost in the United States has no contraindications listed, while the label in Canada does list contraindications: hypersensitivity to iloprost, pregnancy or lactation, risk of hemorrhage (peptic ulcers, trauma, and intracranial hemorrhage), severe coronary heart disease or unstable angina, myocardial infarction (within last 6 months), decompensated cardiac failure, severe arrhythmias, cerebrovascular events (within last 3 months), acute or chronic congestive heart failure, pulmonary hypertension, and congenital or acquired valve defects. Dosage studies have been conducted in hepatic and renal impairment to support recommended dose adjustments listed in labeling in both countries. Iloprost dosing is based on patient tolerance with the high rate of adverse events (23%-73%) resulting in 16%-27% of patients that do not complete the full duration of treatment.^10^^,^^47^^,^^48^ Most adverse reactions are mild—particularly in the setting of risk of loss of digit or limb. The top adverse reactions being: headache, flushing, palpitations, tachycardia, nausea, and hypotension.^10^^,^^47^^,^^48^ Like thrombolytics, no multicenter randomized, peer-review studies have been performed. Currently, one participating center is conducting a randomized control trial to formally evaluate the impact of epoprostenol on frostbite outcomes.^97^

**Figure 7:**
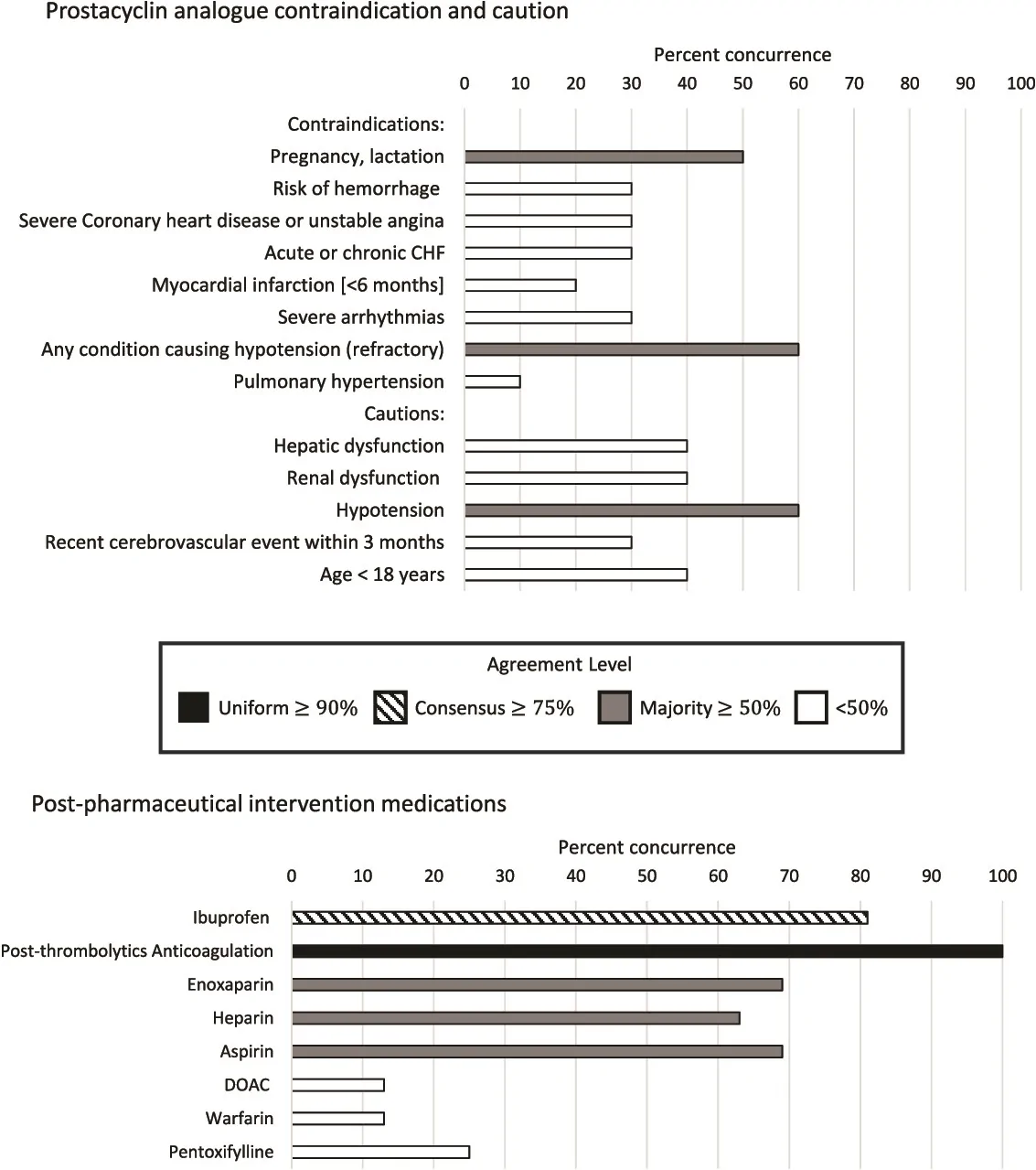
Visual Representation of Protocol Concurrence for Prostacyclin Analog Therapy Contraindications and Cautions (Table S8) and Postpharmaceutical Intervention Medications (Table S9)

*Protocol review* (Table S8): There are currently 10 centers with active protocols for prostacyclin analog use and 2 centers have planned, but not implemented protocols. There are 7 contraindications and 5 cautions all with low agreement. Only hypotension and pregnancy are listed in at least half of protocols. Most protocols stem from the Whitehorse Canada protocol developed by Poole and colleagues, which include the contraindications and cautions listed on the Canadian labeling.^10^ Most center’s protocols initiate IV thrombolytics after the first 1 h of iloprost dosing, so medications are given simultaneously. This mirrors the use of vasodilation in intra-arterial thrombolytic protocols (Figure 7). Epoprostenol contraindications are slightly different, but many are specific to a research study and unrelated to product labeling.^88^

***Opportunities:*** As experience with prostacyclin analog use grows, contraindications and cautions should be modified accordingly. Prostacyclin analogs have vasodilatory, fibrinolytic, and anti-inflammatory properties and use should be monitored in conditions that are at risk for bleeding and hypotension. The recent work by O’Dochartaigh et al. demonstrated a dose response for iloprost.^47^ This, coupled with the high rate of adverse events and nearly a quarter of patients not completing the full duration of treatment, needs future research on protocols to minimize treatment disruption, alleviation of adverse events, and measures to evaluate the total duration of therapy necessary for optimal outcomes. At least one center plans to repeat perfusion assessment after thrombolytics to assess the need for iloprost therapy (personal communication); this is likely due to the high cost and resources required for this pharmacologic intervention in the United States. Most high-volume centers have well-established pathways to initiate thrombolytics early, but how iloprost ultimately becomes implemented at US centers will require a few winters to determine. The prolonged dosing strategy and monitoring requirements should be rigorously evaluated for impact on outcomes (particularly in the setting of thrombolytic usage) and resource allocation. An opportunity exists to use bedside imaging to evaluate both the initiation and the continued need for prostacyclin analogs.

### Posttreatment medications (Table S9)

McCauley et al. called for the administration of aspirin and subsequently ibuprofen for, what was thought at the time, a more specific blockage of the eicosanoid pathway.^40^^,^^101^ This idea has not been replicated and further study demonstrates ibuprofen causes a decrease in both thromboxane (TXA2) and prostacyclin (PGI2) via COX-1 and -2 inhibition.^102^ Field or low resource management of frostbite injury dictates use of ibuprofen or aspirin when other medical interventions are delayed.^53^^,^^55^^,^^103^^,^^104^ The use of adjuvant anticoagulants following thrombolytics for severe frostbite is not well studied.^31^^,^^55^ While anticoagulation in the absence of thrombolytics is not considered efficacious, some form of anticoagulation is generally agreed upon in the management of severe frostbite following thrombolytics.^55^^,^^104^ The recently recanalized vessel is at risk for coagulation and re-clotting until the endothelium has been restored. In cardiac and critical limb ischemia, large studies have shown the benefit of anticoagulation and antiplatelet therapy to prevent further limb ischemia and cardiovascular events.^105–107^ These studies recommend a total duration of 6 months to treat critical limb ischemia.

*Protocol review* (Table S9): There is consensus agreement among centers regarding the use of ibuprofen (81%) and uniform agreement for the use of anticoagulation after thrombolytics (Figure 7). Most use either enoxaparin or heparin. The duration of the anticoagulation varies from days to weeks, though most centers switch to some type of oral management after a period of enoxaparin and/or heparin. The majority of the centers administer aspirin for 1 month. Seven centers transition to or additionally give either warfarin, a direct oral anticoagulant, or pentoxifylline.

***Opportunities:*** Adjuvant pharmacologic treatment for pre and postthrombolytic care needs broader consensus among the centers. As frostbite is an acute process, not due to vascular disease, the treatment may not need prolonged anticoagulation used in ischemic limb disease. While all centers specify the need for anticoagulation, the agent, type, or duration is variable. The literature in ischemic limb continues to evolve with most recommendations showing anticoagulation and antiplatelet useful.^105–107^ For frostbite, one study showed that 1 month of aspirin resulted in similar outcomes compared to warfarin, but this was a small single-center study and no peer reviewed manuscript was ever published.^108^ Ibuprofen is a nonselective prostaglandin inhibitor blocking Cox-1 and -2 and was used to reduce inflammatory mediators in blister fluid of both animals and humans.^50^ Ibuprofen, central to the McCauley protocol, is indicated in all but 3 current protocols. However, ibuprofen is not used outside of frostbite treatment to treat endothelial injury. More study is needed regarding the use of ibuprofen and aspirin treatment for severe frostbite. Research is needed to clarify the role and duration of postthrombolytics anticoagulation and antiplatelet therapy to optimize limb and digit salvage in a safe manner. Additional research should also examine the role of therapies in patients who fail to qualify for thrombolytics or prostacyclin analogs.

### Postfrostbite rehabilitation care (Table S10)

Rehabilitation is central to quality outcomes in burn care. Like burn injury, frostbite involves wounds over joints, potentially limiting range of motion and leading to joint contractures, especially in grafted wounds.^109^ For burn injuries, early therapy and splinting can be helpful in keeping joints mobile and in optimal positions for movement. There is sparse literature regarding physical therapy for frostbite; therefore, treatment is extrapolated from burn care. Only a few studies report disability following frostbite.^1^ Ervasti et al., in a case series 4-11 years after grade 2 frostbite, found that 53% of patients had hypersensitivity to cold, 40% had numbness, and 13% could not work at the same level prior to injury.^110^ In a study of US military cold-injury, 65% of patients reported persistent neurovascular symptoms 6 months following frostbite injury.^111^ In a study of soldiers with frostbite, 50%-89% could not fully return to duty and had to be reassigned or discharged.^112^ Finally, Carlsson et al. found that abnormal thermal and vibration perception thresholds and pain following additional cold exposure continued for years following frostbite in some military personnel.^113^ A single-center retrospective chart review study by Kindt et al. recently compared functional outcomes of frostbite patients treated with thrombolytics or supportive care.^114^ Significantly more lower extremity injured patients treated with thrombolytics were ambulating independently at follow-up, but there was no significant differences in pain, edema, or numbness.^114^ Recently, botulinum toxin injections have been used to reduce long-term sequela following frostbite injury including reduction in cold sensitivity and improvement in blood flow.^115^^,^^116^

**Figure 8:**
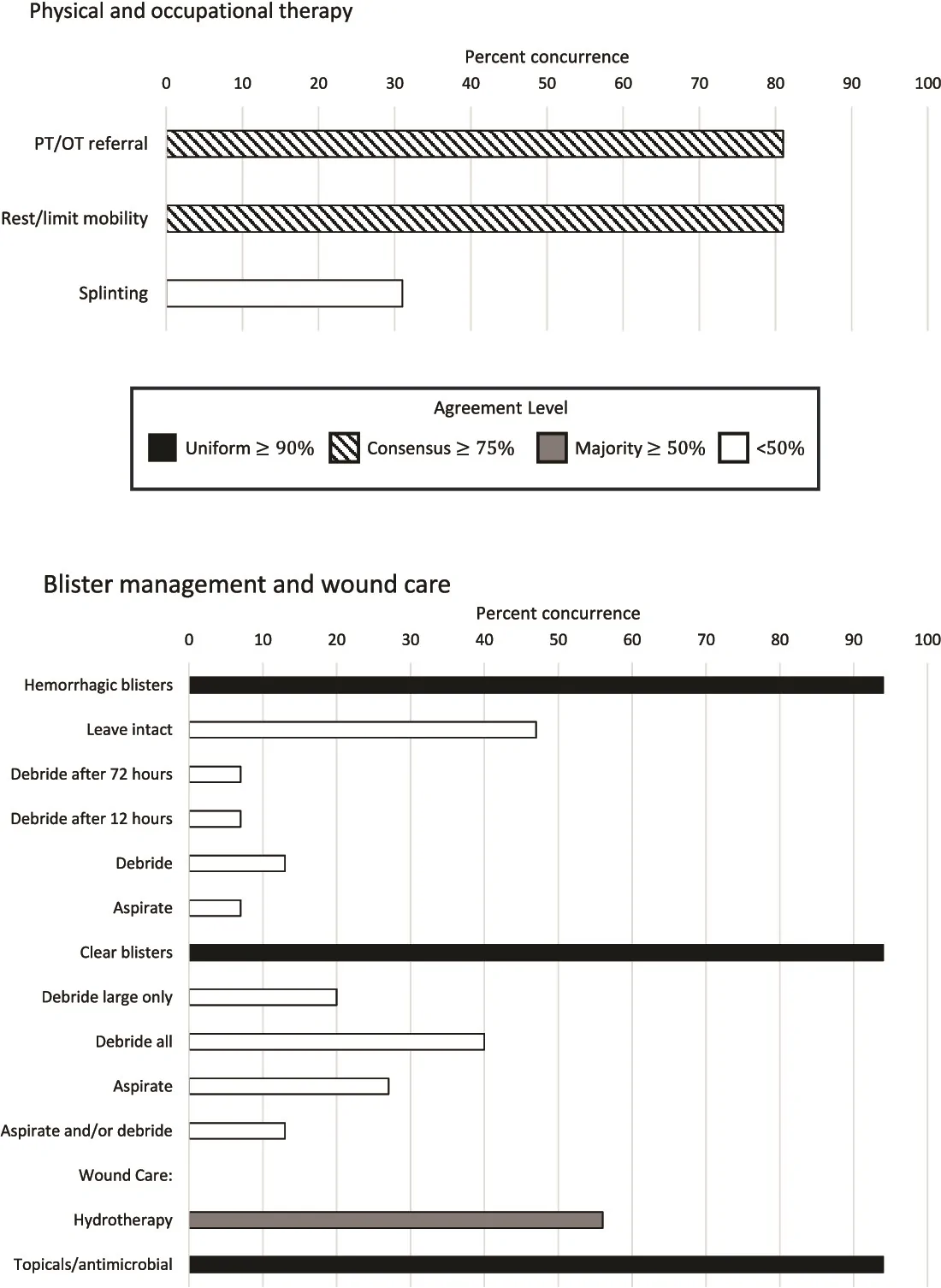
Visual Representation of Protocol Concurrence for Physical and Occupational Therapy (Table S10) and Blister Management and Wound Care (Table S11)

*Protocol review for rehabilitation* (Table S10): The majority of the burn centers specify the need to involve rehabilitation in their frostbite treatment protocols (81%) and early restrictions on weight bearing (81%) (Figure 8).

***Opportunities:*** Long-term outcome data are needed to determine rehabilitation needs during both the acute care and follow-up care of frostbitten patients. In particular, the role of non-weight bearing, splinting, and edema management needs additional study. Other opportunities include the role of the prosthetist and podiatrist in successful outcomes. Evaluation of the use of botulinum toxin to reduce long-term symptoms of frostbite injury are warranted.

### Postfrostbite wound care (Table S11)

Wound care is essential for successful frostbite treatment. Infection occurs in up to 20% of patients either during the acute admission or as a sequela requiring readmission.^4^^,^^7^^,^^8^^,^^117^ Wound care was specified by the McCauley protocol and is similar to that of burn care, differing only in blister management.^40^ Like burn wounds, frostbite results in both partial and full thickness wounds that require routine inspection and cleansing to decolonize them and remove necrotic tissue. Whereas most blisters are unroofed in burn care, hemorrhagic blisters, often associated with severe frostbite and tissue at risk, are left in place to be debrided in a delayed fashion. This treatment is supported by the general opinion that exposing unhealthy tissue may lead to desiccation, colonization, and infection. Treatment also consists of topical antimicrobial therapy and *Aloe vera*, for its anti-thromboxane properties. An ongoing randomized clinical trial is examining *A vera* versus long-acting silver dressings in frostbite wound therapy.^118^ A distinction also must be made between healing frostbite wounds, and ischemic wounds that eventually progress to dry gangrene.

*Protocol review* (Table S11): The protocols uniformly detail blister management for frostbite wound care; however, the methods of management vary widely (Figure 8). Uniform agreement generally supported delaying debridement of hemorrhagic blisters, immediately debriding serous blisters, and on the use of topical agents and antimicrobials. Daily hydrotherapy was mentioned only by a slight majority of centers (56%).

***Opportunities:*** There are opportunities to specify the details of frostbite wound care including blister management, daily care, and topical therapy on wound healing and infection prevention following frostbite injury.

### Surgical planning and management

Although not addressed in the protocols, for completeness this section is added. The historic practice for definitive surgical management of severe frostbite injury is “watchful waiting.” This delayed operative intervention typically occurs 4-6 weeks postinjury to allow complete tissue demarcation, helping to preserve as much limb or digit length and viable tissue as possible.^4^^,^^7^^,^^8^^,^^19^^,^^23^^,^^33–36^^,^^61^^,^^117^^,^^119^ Earlier amputation may increase the risk for re-operation and lead to more proximal amputations.^4^^,^^7^^,^^8^^,^^19^^,^^33–36^^,^^117^ More proximal amputations are associated with greater functional impairments, including difficulty walking, balance issues, and reduced physical capacity.^120–122^ Imaging completed after 8 days postinjury can help with delineation of healthy tissue and bone.^60^^,^^61^^,^^119^^,^^123^ Waiting allows the wounds to mature and the patients to accept the loss of their tissue and limbs. Surgical intervention aims to preserve limb length. This can be done with simple skin grafts to complex flap reconstruction.^23^ Long-term outcomes are not known. As frostbite frequently impacts patients with limited expendable income, this may limit access to prosthetics, appropriate footwear, and rehabilitation services, which are often out of pocket expenses in the United States. While typically done in a delayed manner, the timing of amputation is not protocolized, but we felt inclusion of this information may be beneficial for the readers.

### Synthesis of standard frostbite protocols

**Figure 9:**
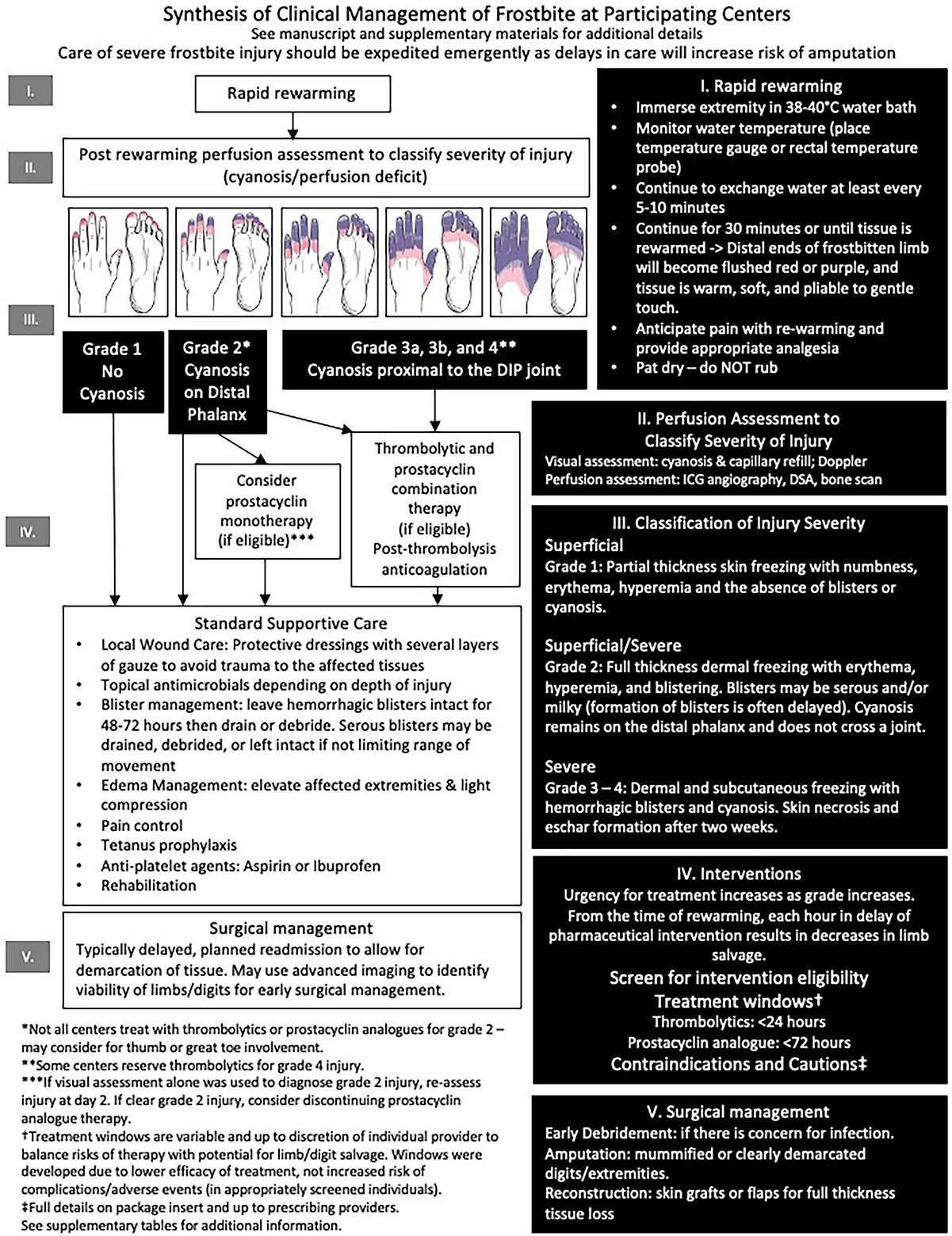
Synthesis of Clinical Management of Frostbite. Figure for use with Supplementary File 1 for more detailed information.

The summation of this review and expert opinion includes detailed synthesis protocol in Figure 9 and File S1.

## DISCUSSION

This is the first manuscript from the FROST study, providing a synthesis and evaluation of frostbite management protocols across 16 high-volume centers. It addresses a current gap in the literature by offering granular detail on clinical practices that existing clinical practice guidelines, developed through formal consensus processes, do not fully capture.^31^^,^^55^ Analysis of these institutional protocols show (1) the emergence of agreement between protocols despite the lack of robust scientific literature and (2) an opportunity to update and standardize frostbite treatment. Though research is still lacking to support robust guidelines, expert opinion and extrapolation from similar disease processes can be used to standardize care. Standardization of protocols will help build the body of knowledge needed to improve frostbite care. Unfortunately, many patients delay seeking care for their injuries and arrive outside treatment windows resulting in high rates of amputation and long-term disability. Continued focus on education of the public, community services, emergency, and medical providers can help disseminate knowledge and emphasize the time sensitive nature of frostbite treatment.

There are also emerging treatment strategies and diagnostic modalities in the care of frostbite. The role of bedside perfusion imaging and use of dopplers to diagnose the severity of frostbite is an area of opportunity. The use of ICG angiography has growing evidence and is increasingly used for more rapid diagnosis of vascular compromise in severe injury. Greater use of these modalities could help delineate salvage rates more precisely. Grading using bedside imaging techniques could also be compared to the visual method to determine agreement. As frostbite is time sensitive, half of centers have protocols for remote delivery of thrombolytics. Remote delivery of thrombolytics is routine for stroke care and should become routine for appropriately screened frostbite patients. Tenecteplase offers a shorter dose option for thrombolysis. In other disease states, tenecteplase has shown either equivalent or superior efficacy.^105–107^ Evaluating the impact of prostacyclin analogs (iloprost and epoprostenol) on outcomes in US frostbite patients is urgently needed. Acute frostbite wound care is similar to that of burns and other wounds that benefit from daily cleansing to decrease bacterial counts, remove necrotic tissue, apply topical ointments, and observe for infection. Centers also uniformly recognize the importance of blister management, but lack agreement on specific treatment and subsequent wound care. At this point in time, there is a lack of scientific support for any method, so it is generally up to the discretion of each specific provider. The efficacy and duration of the application of *A vera* ointment, a prostaglandin inhibitor lacking antimicrobial properties, likewise needs more research. As these wounds are painful and often involve areas of function and joints, rehabilitation is important to maintain function. More study on the impact of rehabilitation for frostbite patients is urgently needed and would be helpful to guide institutional protocols.

In conclusion, there is relative consensus among North American centers regarding the acute treatment of frostbite patients. This manuscript provides a synthesis of protocols for the acute management of frostbite injury. Further advancement will require more research to fine tune frostbite care. In addition, education regarding prevention and the need for emergent treatment is needed for both the public and the medical community. This synthesized protocol may serve as a tool for centers to develop their own standard protocol to facilitate care for frostbite patients.

## Supplementary Material

Supplementary_material_irag111

## Notes

Evidence: Level 5 /expert consensus.

Author contributions: Rachel M. Nygaard (Conceptualization [lead], Data curation, Formal analysis [equal], Funding acquisition, Investigation, Methodology, Project administration, Resources, Supervision [lead], Validation [equal], Visualization [lead], Writing—original draft [equal], Writing—review & editing [lead]), Emily Colonna (Data curation [equal], Investigation, Project administration, Writing—review & editing [supporting]), Alexander Poole (Data curation, Formal analysis, Investigation, Methodology, Writing—review & editing [supporting]), Lee D. Faucher (Data curation, Formal analysis, Investigation, Methodology, Writing—review & editing [supporting]), Rosemary E. Paine (Data curation, Formal analysis, Investigation, Methodology, Writing—review & editing [supporting]), Naveen Sangji (Data curation, Formal analysis, Investigation, Validation, Writing—review & editing [supporting]), Alexandra M. Lacey (Data curation, Formal analysis, Investigation, Validation, Writing—review & editing [supporting]), Dhaval Bhavsar (Data curation, Formal analysis, Investigation, Validation, Writing—review & editing [supporting]), Kristin Colling (Data curation, Formal analysis, Investigation, Validation, Writing—review & editing [supporting]), Alexis Armour (Data curation, Formal analysis, Investigation, Validation, Writing—review & editing [supporting]), Irma Fleming (Data curation, Formal analysis, Investigation, Validation, Writing—review & editing [supporting]), Arek J. Wiktor (Data curation, Formal analysis, Investigation, Validation, Writing—review & editing [supporting]), and Lucy Wibbenmeyer (Conceptualization, Data curation, Formal analysis [equal], Funding acquisition [supporting], Investigation, Methodology, Project administration, Resources [equal], Supervision, Validation, Visualization [supporting], Writing—original draft [equal], Writing—review & editing [supporting])

Funding: This study is funded by the Military Burn Research Program of the Congressionally Directed Medical Research Programs for the Department of Defense (HT9425-24-1-0273).

Conflict of interest statement: R.M.N.: Principal Investigator for the Military Burn Research Program FROST study (research funding); contract science writing for Civibio (2022). E.C.: None to disclose. A.P.: None to disclose. LF: None to disclose. R.P.: None to disclose. N.S.: American College of Surgeons Faculty Research Fellowship (2024-2026); salary support from the Michigan Department of Health and Human Services (MDHHS) grant to the Regents of the University of Michigan for the State Burn Coordinating Center (funding unrelated to this study). A.L.: None to disclose. D.B.: Contract research as part of a multicenter trial from PolyNovo, Vericel, and Spectral AI (funding unrelated to this study). K.C.: None to disclose. A.A.: None to disclose. I.F.: None to disclose. A.W.: Military Burn Research Program, Military Infectious Disease Research Program, and Combat Casualty Care Research program (research funding unrelated to this study). L.W.: Co-investigator for the Military Burn Research Program FROST study (research funding); contract research as part of a multicenter trial from PolyNovo, Spectral AI, Skingenix, and Kerecis; consultant for PolyNovo and Vericel (funding unrelated to this study).
