# Supplementary material for: Synthesis of Protocols for Frostbite Care From the FROST Study Sites
Source: J Burn Care Res. 2026 Jul 1;47(5):1405–25. doi: 10.1093/jbcr/irag111 (PMC13551621; doi:10.1093/jbcr/irag111)
Supplement: Supplementary file 1 [file supplementary_material_irag111.zip › FROST_protocol_supplementary_tables_041526_irag111.docx]

Supplementary Table 1: Rewarming strategies

| Management Aspect | Percent concurrence | A | B | C | D | E | F | G | H | I | J | K | L | M | N | O | P |
| --- | --- | --- | --- | --- | --- | --- | --- | --- | --- | --- | --- | --- | --- | --- | --- | --- | --- |
| Rapid Rewarming | 94 | x | x | x | x | x | x |  | x* | x | x | x | x | x | x | x | x |
| Temp (C) | 93 | 37-39 | 37-39 | 38-40 | 40 | 40 | 40 |  |  | 42 | 38-42 | 38-42 | 38-40 | 36 | 39 | 40 | 40-42 |
| Duration (min) | 87 | 20-40 | 30 | 30-45 | 20-40 |  | 10-15 |  |  | 30 | 15-20 | 15-30 | 20-60 | 30-45 | 30 | 30 | 20-30 |
| Protocol specifies: |  |  |  |  |  |  |  |  |  |  |  |  |  |  |  |  |  |
| How to set up | 47 | x |  | x |  |  |  |  |  | x |  | x | x | x |  | x |  |
| How to regulate temp | 40 | x |  |  |  |  | x |  |  | x |  |  | x | x |  | x |  |
| Describes how limb/digit should appear when warming is complete | 67 | x |  | x | x |  | x |  |  | x |  |  | x | x | x | x | x |
| Pain management noted | 87 | x | x | x | x |  | x |  | x | x | x | x | x | x |  | x | x |
| Other rewarming methods | 56 | x | x | x | x |  |  | x |  | x |  |  | x | x |  | x |  |
| Warm IV fluids | 50 | x | x | x | x |  |  |  |  | x |  |  | x | x |  | x |  |
| Forced air-warming system | 38 |  | x |  | x |  |  | x |  | x |  |  | x |  |  | x |  |
| Warm blankets | 44 |  | x | x | x |  |  | x |  | x |  |  | x | x |  |  |  |
| Heat Lights | 13 |  | x |  | x |  |  |  |  |  |  |  |  |  |  |  |  |

*Mentions, but lacks details

Supplementary Table 2. Perfusion diagnostics used

| Management Aspect | Percent concurrence | A | B | C | D | E | F | G | H | I | J | K | L | M | N | O | P |
| --- | --- | --- | --- | --- | --- | --- | --- | --- | --- | --- | --- | --- | --- | --- | --- | --- | --- |
| Perfusion Imaging Before Thrombolytics | 75 |  | +/- | +/- | x | x | x | x |  | x |  | x | x | * |  | x | x |
| Perfusion Imaging After Thrombolytics | 75 |  | x | x | +/- | x |  | x | x | x | x | x | x | x |  | x |  |
| Perfusion assessment |  |  |  |  |  |  |  |  |  |  |  |  |  |  |  |  |  |
| Visual | 88 | x | x | x | x | * | x | x | x |  |  | x | x | x | x | x | x |
| Doppler | 56 |  | x | x | +/- |  | x | x |  |  | x |  | x |  |  | x | x |
| ICG angiography (LUNA/SPY) | 38 |  |  |  | x | x |  |  |  | x |  |  | x |  |  | x | x |
| Angiography | 44 |  | x |  |  |  | x | x |  | x | x |  | x | x |  |  |  |
| Tc99 scan | 63 |  | x | x |  | x | x |  | x | x | x | x |  | x |  |  | x |

+/- consider, * only with IA delivery

Supplementary Table 3. Timing and severity of injury for thrombolytic intervention

|  | Percent concurrence | A | B | C | D | E | F | G | H | I | J | K | L | M | N | O | P |
| --- | --- | --- | --- | --- | --- | --- | --- | --- | --- | --- | --- | --- | --- | --- | --- | --- | --- |
| Window for treatment (hours) |  |  |  |  |  |  |  |  |  |  |  |  |  |  |  |  |  |
| Thrombolytics: IV | 12h = 14  24h =79  48h = 7 | 24 | 24 | 24 | 12 | ~12 | 24 |  | 24 | 24 |  | 24 | 24 | 24 | 24 | 48 | 24 |
| Thrombolytics: IA | 24h= 86  48h = 14 |  | 24 |  |  |  | 24 | 48 |  | 24 | 24 |  | 24 | 24 |  |  |  |
| **Thrombolytics: IV** | 88 | x | x | x | x | x | x |  | x | x |  | x | x | x | x | x | x |
| Perfusion deficit on imaging | 64 |  | x | x | x | x |  |  |  | x |  | x |  | x |  | x | x |
| Visual grade 2 | 14 |  |  | x |  |  |  |  |  |  |  | x |  |  |  |  |  |
| Visual grade 3 | 79 | x | x | x | x | x | x |  | x | x |  | x | x | x |  |  |  |
| Visual grade 4 | 93 | x | x | x | x | x | x |  | x | x |  | x | x | x | x | x |  |
| Doppler (lack of pulses) | 43 |  | x | x |  |  | x |  |  |  |  |  |  | x |  | x | x |
| Severe frostbite with clear evidence of frozen tissue | 43 |  |  | x |  | x |  |  | x |  |  |  | x | x |  | x |  |
| No improvement after rapid rewarming (clinical judgement) | 57 |  |  | x |  | x | x |  | x |  |  |  | x | x |  | x | x |
| **Thrombolytics: IA** | 44 |  | x |  |  |  | x | x |  | x* | x |  | x | x |  |  |  |
| Perfusion deficit on imaging | 71 |  | x |  |  |  |  | x |  | x |  |  | x | x |  |  |  |
| Visual grade2 | 14 |  |  |  |  |  |  | x |  |  |  |  |  |  |  |  |  |
| Visual grade3 | 71 |  | x |  |  |  | x | x |  | x |  |  |  | x |  |  |  |
| Visual grade 4 | 71 |  | x |  |  |  | x | x |  | x |  |  |  | x |  |  |  |
| Doppler (lack of pulses) | 71 |  | x |  |  |  | x | x |  |  | x |  |  | x |  |  |  |

* Used when isolated to UE

Supplementary Table 4. Dosage of thrombolytic administration

| thrombolytics | Percent concurrence | A | B | C | D | E | F | G | H | I | J | K | L | M | N | O | P |
| --- | --- | --- | --- | --- | --- | --- | --- | --- | --- | --- | --- | --- | --- | --- | --- | --- | --- |
| IV | 88 | x | x | x | x | x | x |  | x | x |  | x | x | x | x | x | x |
| IV alteplase | 93 |  |  |  |  |  |  |  |  |  |  |  |  |  |  |  |  |
| Alteplase bolus |  | 1 | 2 | 3 | 1 | 1 | 2 |  | 3 |  |  | 4 | 1 | 4 | 1 | 3 | 4 |
| Alteplase infusion |  | 5 | 6 | 5 | 5 | 5 | 5 |  | 5 |  |  | 7 | 6 | 7 | 5 | 5 | 5 |
| Alteplase total dose max (mg) |  | 100 | 100 | 100 | 100 | 100 | 99.75 |  | 100 |  |  | 100 | 105 | 100 | 100 | 100 |  |
| IV tenecteplase | 36 |  |  |  |  |  |  |  |  |  |  |  |  |  |  |  |  |
| Tenecteplase dose (mg/kg) |  | 0.25 |  |  |  |  |  |  |  | 0.25 |  |  | 0.25 |  | 0.25 | 0.25 |  |
| Tenecteplase max dose (mg) |  | 25 |  |  |  |  |  |  |  | 25 |  |  | 25 |  | 25 | 25 |  |
| IA | 44 |  | x |  |  |  | x | x |  | x | x |  | x | x |  |  |  |
| IA alteplase |  |  |  |  |  |  |  |  |  |  |  |  |  |  |  |  |  |
| Alteplase infusion | 100 |  | 8 |  |  |  | 9 | 10 |  | 10 | 11 |  | 12 |  |  |  |  |
| Alteplase total dose max (mg) |  |  | Per IR |  |  |  | 99.75 | 100 |  | Per IR | Per IR |  | Per IR | Per IR |  |  |  |

Footnote:

1. 0.15mg/ kg over 15 min
2. 40-94kg: 0.15 mg/kg/ 15 min. 95kg+: 14.2 mg/kg/15 min
3. 0.15 mg/kg/2 min
4. 0.15mg/kg
5. 0.15mg/kg/hour for 6 hours
6. 0.15 mg/kg/hour, for 6 hours. 95kg+: 14.2 mg/hour, for 6 hours
7. <95 kg: Bolus dose = 0.15 mg/kg (no max dose for bolus), Infusion = 0.9 mg/kg at a rate of 0.15 mg/kg/hour over 6 hours  >95 kg Bolus dose = 0.15 mg/kg (no max dose for bolus)  Infusion = 0.9 mg/kg (adjusted to a total of 100 mg including the bolus) , at a rate of 0.15 mg/kg/hour
8. 0.15mg/kg/hour over 4-6 hours
9. 0.5 mg/hour per limb with max of 2 mg/hr. not to exceed 48 hours
10. Bolus – 0.15 mg/kg, infusion – 1 mg/hour (max 48 hours)
11. 0.5mg/kg in individual limbs
12. 1 mg/hour (dose divided by number of affected extremities)

Supplementary Table 5. Dosage of prostacyclin analogue administration

| Management Aspect | Percent concurrence | A | B | C | D | E | F | G | H | I | J | K | L | M | N | O | P |
| --- | --- | --- | --- | --- | --- | --- | --- | --- | --- | --- | --- | --- | --- | --- | --- | --- | --- |
| Prostacyclin Analogue Window (hours) | 72: 100 | 72 | 72 | 72 |  | 48* | 72 |  |  | 72 |  | 72 | 72* | 72 | 72 | 72 | 72 |
| **Prostacyclin analogue** | 63 | x | x | x |  | * | x |  |  | x |  | x | * | x | x | x | x |
| Perfusion deficit on imaging | 40 |  |  | x |  | x |  |  |  | x |  | x |  |  |  |  | x |
| Visual grade 2 | 70 | x | ** | x |  | *** |  |  |  |  |  | x | x | x | ** | x |  |
| Visual grade 3 | 100 | x | x | x |  | x | x |  |  | x |  | x | x | x | x | x | x |
| Visual grade 4 | 100 | x | x | x |  | x | x |  |  | x |  | x | x | x | x | x | x |
| Doppler (lack of pulses) | 40 |  |  |  |  |  | x |  |  | x |  |  |  | x |  |  | x |
| Duration (days) | 5: 60  8: 40 | 5 | 5 | 5 |  | 5 | 8 |  |  | 8 |  | 8 | 5 | 5 | 5 | 5 | 8 |
| Dosage |  | 1 | 1 | 2 |  | 5 | 3 |  |  | 5 |  | 3 | 3 | 3 | 4 | 4 | 5 |

*plan, but not currently implemented, so not included in summary statistics **reassess at 24 hours and if clear grade 2, discontinue. ***discretion of provider

Footnote

1. Iloprost: 40-50kg: 2-6 mcg/hour (10-30 mL/hour), titrated IV, for 6 hours. 51-75kg: 2-8 mcg/hour (10-40 mL/hour), titrated IV, for 6 hours. 75+kg: 2-10 mcg/hour (10-50 mL/hour), titrated IV, for 6 hours.
2. Epoprostenol: 1-8 ng/kg/min x 1 hr. Titrate IV as tolerated for 8 hours.
3. Iloprost: Days 1-3: Initial IV: 0.5 ng/kg/min; increase infusion rate by 0.5 ng/kg/min every 30 minutes as tolerated up to a maximum of 2 ng/kg/min. Total duration: 6 hours per day. Days 4-5: Start infusion at highest tolerated dose from previous day; adjust rate as needed based on tolerability (titrating by 0.5 ng/kg/min in either direction every 30 minutes as tolerated up to a maximum of 2 ng/kg/min). Total duration: 6 hours per day. Escalating to the max on day 3.
4. Iloprost: Dilute 50 mcg (0.5 mL) in Dextrose 5% (D5W) 250 mL bag for a final concentration of 0.2 mcg/mL. IV infusion at 10 mL/hour and increase infusion rate by 10 mL/hour every 30 minutes to a maximum of: q 30 mL/hour for patients 40-50 kg; q 40 mL/hour for patients 51-74 kg; q 50 mL/hour for patients 75 kg or more
5. Iloprost: <65 and no renal/hepatic compromise, start at 0.5ng/kg/min and titrate up by 0.5ng/kg/min every 30 minutes up to 2ng/kg/min as tolerated. If > or = 65 or with renal/hepatic compromise, start at 0.25ng/kg/min and titrate as above.

Supplementary Table 6. Contraindications to thrombolytics

| Contraindications | Percent concurrence | A | B | C | D | E | F | G | H | I | J | K | L | M | N | O | P |
| --- | --- | --- | --- | --- | --- | --- | --- | --- | --- | --- | --- | --- | --- | --- | --- | --- | --- |
| Time from rewarming | >12: 12.5%  >24: 75%  >48: 12.5% | >24 | >24 | >24 | >12 | ~>12 | >24 | >48 | >24 | >24 | >24 | >24 | >24 | >24 | >24 | >48 | >24 |
| Freeze thaw cycles | 56 |  | x | x |  |  |  |  |  | x | x | x | x | x |  | x | x |
| *>48 hours cold exposure* | 44 | x | x |  |  |  |  | x |  |  | x |  |  | x |  | x | * |
| Acute systemic hypothermia being actively treated | 25 | x |  |  |  | x |  |  |  |  |  |  | x | x |  |  |  |
| History of ischemic stroke (<2 months to <3 months) | 88 | x | x | x | x |  | x | x | x | x |  | x | x | x | x | x | x |
| Intracranial or intraspinal surgery (<2 months to <3 months) | 75 | x | x | x | x | x |  |  |  | x | x | x | x | x | x | x |  |
| Intracranial bleed or subarachnoid hemorrhage (<1 month) | 75 | x | x | x | x |  |  | x | x | x | x |  | x | x | x | x |  |
| Significant closed head trauma or facial trauma (<1 month to <2 months | 88 | x | x | x | x | x | x | x | x | x |  | x | x | x | x | x |  |
| Known intracranial arteriovenous malformation, neoplasm, or aneurysm | 81 | x | x | x | x | x | x | x | x |  |  | x | x | x | x | x |  |
| Suspected aortic dissection | 50 | x |  | x | x | x | x |  | x |  |  |  |  | x |  | x |  |
| Uncontrolled unresponsive hypertension | 75 | x |  | x |  |  | x |  | x | x | x | x | x | x | x | x | x |
| Active bleeding, with the exception of menses | 88 | x | x | x | x | x | x |  | x | x | x | x | x | x | x | x |  |
| Recent major trauma or surgery | 81 |  | x | x | x |  | x | x | x | x | x |  | x | x | x | x | x |
| prolonged CPR | 63 |  | x |  | x | x |  |  | x | x | x |  | x | x |  | x | x |
| <18 or *Weight < 40 kg* | 13 |  |  |  |  |  | x |  |  |  |  |  |  | x |  |  |  |
| Pregnancy | 81 | x | x | x | x | x | x |  | x | x | x |  |  | x | x | x | x |
| Known bleeding diathesis:  ● Platelet count <100,000K  ● Heparin administration with elevated PTT  ● Current use of warfarin with INR>1.7  ● Current use of direct thrombin inhibitors or direct factor xa inhibitors | 88 | x | x | x | x | x | x | x | x | x |  | x | x | x | x | x |  |
| Inability to obtain history or consent due to altered mental status | 63 | x |  | x | x | x | x |  | x | x | x ** |  |  | x |  |  | x |

 Note: * greater than 24 hours; **drug/alcohol/intoxication is a contraindication.

Supplementary Table 7. Cautions for thrombolytics

| **Caution** | Percent concurrence | A | B | C | D | E | F | G | H | I | J | K | L | M | N | O | P |
| --- | --- | --- | --- | --- | --- | --- | --- | --- | --- | --- | --- | --- | --- | --- | --- | --- | --- |
| Cautions listed | 81 | x | x | x | x | x | x | x | x |  |  | x | x | x | x | x |  |
| Greater than 24 hours of cold exposure | 19 |  |  |  | x |  | x |  |  |  |  |  |  |  |  | x |  |
| Warm ischemia time greater than 6 hours | 19 |  |  |  | x |  | x |  |  |  |  |  |  |  |  | x |  |
| Multiple freeze thaw cycles | 63 | x | x | x | x | x | x | x |  |  |  |  | x | x |  | x |  |
| Prolonged (>10 min) CPR | 69 | x | x | x | x | x | x | x |  |  |  |  | x | x | x | x |  |
| Serious trauma [concurrent - within 3 months] | 63 | x | x | x | x | x | x |  |  |  |  | x | x | x |  | x |  |
| Non-compressible vascular punctures | 44 | x | x | x |  |  | x |  | x |  |  |  |  | x |  | x |  |
| Major surgery [2 days to 3 months] | 69 | x | x | x | x | x | x |  | x |  |  |  | x | x | x | x |  |
| History of gastrointestinal or other hemorrhage [2 weeks to 3 months] | 69 | x | x | x | x |  | x | x | x |  |  |  | x | x | x | x |  |
| Myocardial infarction [<3 months] | 19 | x | x |  |  |  |  |  |  |  |  |  |  |  |  | x |  |
| Abdominal aortic aneurysm or aortic dissection | 19 |  | x |  |  |  |  |  |  |  |  |  |  | x |  | x |  |
| Prior intracranial hemorrhage, known structural intracranial process, intracranial neoplasm (<3 months). | 63 |  | x | x |  | x | x |  | x |  |  | x | x | x | x | x |  |
| History of ischemic stroke | 56 |  | x | x |  | x | x | x |  |  |  |  | x | x | x | x |  |
| Hypercoagulable states [platelets < 100,000 mm3, INR greater than 1.7, LMWH (therapeutic dose within 24 hours) and Direct Oral Anticoagulants (unless laboratory tests are normal or no dose for > 48 hours), Elevated aPTT (>80)] | 50 |  | x | x | x |  | x |  | x |  |  |  | x | x |  | x |  |
| Uncontrolled hypertension that does not respond to treatment | 50 |  | x | x | x | x | x | x |  |  |  |  | x | x |  |  |  |
| Active malignancy | 13 |  | x |  |  |  |  |  |  |  |  |  |  | x |  |  |  |
| Pregnancy or postpartum less than 6 weeks | 75 | x | x | x | x | x | x | x | x |  |  |  | x | x | x | x |  |
| Dementia/altered mental status | 31 |  |  | x |  |  | x |  |  |  |  |  |  | x | x | x |  |
| Advanced age (>75) | 31 |  |  | x | x |  | x |  |  |  |  |  |  | x |  | x |  |
| Low age (<14) | 19 |  |  |  |  |  | x |  |  |  |  |  |  | x |  | x |  |

Supplementary Table 8. iloprost contraindications and cautions

| **Iloprost** | Percent concurrence | A | B | C | D | E^ | F | G | H | I | J | K | L^ | M | N | O | P |
| --- | --- | --- | --- | --- | --- | --- | --- | --- | --- | --- | --- | --- | --- | --- | --- | --- | --- |
| **Contraindications** |  | x | x* | x |  | x | x |  |  | x* |  | x | x | x | x | x | x* |
| Pregnancy, lactation | 50 | x |  | x |  | x |  |  |  |  |  |  | x | x | x | x |  |
| Risk of hemorrhage (active peptic ulcers, trauma, intracranial hemorrhage) | 30 |  |  |  |  | x |  |  |  |  |  |  | x | x | x | x |  |
| Severe Coronary heart disease or unstable angina | 30 |  |  |  |  | x |  |  |  |  |  |  | x | x | x | x |  |
| Acute or chronic CHF | 30 |  |  | x |  | x |  |  |  |  |  |  | x |  | x | x |  |
| Myocardial infarction [<6 months] | 20 |  |  |  |  | x |  |  |  |  |  |  | x |  | x | x |  |
| Severe arrhythmias | 30 |  |  |  |  | x |  |  |  |  |  |  | x | x | x | x |  |
| Any condition causing hypotension (refractory) | 60 | x |  | x |  |  | x |  |  |  |  | x |  | x |  |  |  |
| Pulmonary hypertension | 10 |  |  | x |  |  |  |  |  |  |  |  |  |  |  |  |  |
| **Cautions** |  |  |  |  |  |  |  |  |  |  |  |  |  |  |  |  |  |
| Hepatic dysfunction with Child’s Pugh score of B or C and in patients with renal failure requiring dialysis | 40 | x |  |  |  | x |  |  |  |  |  |  | x | x | x | x |  |
| Renal dysfunction with an eGFR <30 | 40 | x |  |  |  |  |  |  |  |  |  |  |  | x | x | x |  |
| Hypotension | 60 | x |  |  |  | x | x |  |  |  |  | x | x | x | x | x |  |
| Recent cerebrovascular event or transient ischemic attack, stroke within 3 months | 30 |  |  |  |  | x |  |  |  |  |  |  |  | x | x | x |  |
| Age < 18 years | 40 | x |  |  |  |  |  |  |  |  |  |  | x | x | x | x |  |

*Noted contraindications but not detailed in protocol; ^planned protocols, not included in descriptive statistics.

Supplementary Table 9. Post-treatment medication dose and duration

| Management Aspect | Percent concurrence | A | B | C | D | E | F | G | H | I | J | K | L | M | N | O | P |
| --- | --- | --- | --- | --- | --- | --- | --- | --- | --- | --- | --- | --- | --- | --- | --- | --- | --- |
| Ibuprofen (dose/duration) | 81 | 400 mg QID  7 days | 600 mg  QID  5 days |  | 600mg QID | 600mg TID | 600mg TID | 600mg QID | 400-600 mg QID |  | 400-600 mg QID | 6 mg/kg/ BID  14 days | 400 mg BID | 600mg QID, up to 4 weeks | 600mg QID | 800 mg on arrival; 400 mg QID |  |
| Post-thrombolytics Anticoagulation | 100 | x | x | x | x | x | x | x | x | x | x | x | x | x | x | x | x |
| Enoxaparin (1 mg/kg subCUTANEOUS every 12 hours (days) | 69 | 3 |  | Up to 14 | 14 | 7 | 14 - 28 |  | 3 | 7 |  |  | 7 | 3 - 5 | 3 | 7 |  |
| Heparin (80 units/kg/dose bolus – 0-45 units/kg/hr iv titrated -therapeutic levels) (days) | 63 | * | * |  |  |  | 7 - 28 | 3 - 4 | 3 | 1 | * | * |  | 3 - 5 |  |  | 3 - 5 |
| Aspirin Use (81 mg daily) | 69 | 4 weeks ** |  | Up to 1 month | * | 1 month |  |  | 1 month | 30 days |  | * | 30 days | 4 weeks ** |  | *** | 325 mg daily |
| DOAC (days) | 13 |  |  |  |  |  | * |  |  |  |  |  | 7 |  |  |  |  |
| Warfarin (days) | 13 |  |  |  |  |  | * | 30 |  |  |  |  |  |  |  |  |  |
| Pentoxifylline | 25 | 400mg TID |  |  |  |  |  |  |  |  | * | * |  | 6-8 weeks or until surgery |  |  |  |

Note: *Not specified but listed in protocol; ** after enoxaparin or heparin, *** Post thrombolytics until wound closed or at least a month.

Supplementary Table 10. PT/OT

| Management Aspect | Percent concurrence | A | B | C | D | E | F | G | H | I | J | K | L | M | N | O | P |
| --- | --- | --- | --- | --- | --- | --- | --- | --- | --- | --- | --- | --- | --- | --- | --- | --- | --- |
| PT/OT referral | 81 | x | x | x | x | x | x | x |  | x |  |  | x | x | x | x | x |
| Rest/limit mobility | 81 | 1 | 2 | 2 | 3 | 4 | 5 | 3 |  | 7 |  |  | 6 | 8 | 9 | 10 | 11 |
| Splinting | 31 | x |  | x | x |  |  |  |  |  |  | x |  |  |  | x |  |

Note:

1. Limit for 72 hours
2. Limit non weight bearing (NWB) in the immediate 24 to 48 hours depending on depth of injury/TPA use
3. Elevation, foam off loading boots
4. Limit for 48 hours
5. WBAT if partial thickness injury. Non weight bearing (NWB) 72 hours if injury indeterminate and/or deeper.
6. No mobility while on lytics
7. No restrictions
8. Limit mobility for 24-48 all types
9. Limit mobility for all/Consult therapy for splinting
10. Non weight bearing (NWB) in the immediate 24 – 72 hours
11. Elevation/weight bearing restrictions 72 hours

Supplementary Table 11. Blister management and wound care

| Management Aspect | Percent concurrence | A | B | C | D | E | F | G | H | I | J | K | L | M | N | O | P |
| --- | --- | --- | --- | --- | --- | --- | --- | --- | --- | --- | --- | --- | --- | --- | --- | --- | --- |
| Hemorrhagic blisters | 94 | 1 | 1 | 1 |  | 2 | 3 | 1 | 4 | 5 | 1 | 1 | 6 | 1 | 7 | 8 | 9 |
| Clear blisters | 94 | 5 | 6 | 10 |  | 10 | 11 | 12 | 10 | 5 | 13 | 1 | 14 | 15 | 9 | 8 | 9 |
| Wound Care |  |  |  |  |  |  |  |  |  |  |  |  |  |  |  |  |  |
| Hydrotherapy | 56 |  |  | daily |  | BID |  |  | daily | daily |  | daily | daily | daily | daily | daily |  |
| Topicals/antimicrobial | 94 | 16, 17 | 17 | 18, 23 | 17 | 18, 23 | 18, 23 |  | 19 | 17 | 17 | 18, 24 | 20, 23, 24, 25 | 18, 23, 25 | 18 | 21, 23, 25 | 22 |

1. Leave intact
2. Debride 48 hours
3. Debride after 72 hours.
4. Debride after 12 hours
5. debride large blisters
6. debride within 7 days or before discharge
7. Consult surgery
8. Gentle debridement of blisters
9. Aspirate/decompress but do not debride
10. Debride immediately
11. debride and remove serous blisters. Small blisters (around ~1cm or less) without significant fluid may be left intact regardless of their nature.
12. Debride, cover with non adherent anti microbial dressing and gauze
13. Drain and place in aloe vera and/or soft silicone foam dressing
14. Drain after lytics, debride within 7 days or before discharge
15. May debride or drain if impairing ROM.
16. Aloe or iodine-based
17. Provider dependent
18. Aloe
19. silver sulfadiazine topical cream to open - Moisturizer to closed
20. thin bacitracin or betadine paint with bismuth tribromophenate petrolatum gauze
21. Bacitracin with cuticerin
22. bacitracin/polymyxin B or silver sulfadiazine topical cream
23. Bacitracin
24. Betadine
25. Silver sulfadiazine topical cream
