# Supplementary material for: Synthesis of Protocols for Frostbite Care From the FROST Study Sites
Source: J Burn Care Res. 2026 Jul 1;47(5):1405–25. doi: 10.1093/jbcr/irag111 (PMC13551621; doi:10.1093/jbcr/irag111)
Supplement: Supplementary file 1 [file supplementary_material_irag111.zip › FROST_supplementary_file_1_synthesis_protocol_041526_irag111.docx]

**Supplementary File 1: Synthesis of Clinical Management of Frostbite at Participating Centers**

Additional details provided in manuscript, supplementary materials, and supplementary figure 1.

**I. Initial Assessment and Rewarming**

- Care of the severe frostbite patient needs to be expedited emergently as delays in care will increase the likelihood of limb and digit loss.
- Rapid rewarming of frozen extremity
- Remove all clothing from patient
- Warmed IV fluids, warm blankets, forced warm air system, increase room temperature
- Immerse frozen extremity(ies) in ~38-40°C water bath (place temperature gauge or rectal temperature probe in warm water to monitor water temperature)
  - Continue to exchange water at least every 5-10 minutes to keep temperature of water between 38-40°C
  - Continue for 30 minutes or until tissue is rewarmed
    - distal ends of frostbitten limb will become flushed red or purple, and tissue is warm, soft, and pliable to gentle touch.
  - Anticipate pain with re-warming and provide appropriate analgesia
  - Pat dry – do NOT rub
  - Photograph all injured areas after rewarming

**II.** **Perfusion assessment** - All imaging should be performed after extremity is RE-WARMED.

Both visual assessment of perfusion and formal perfusion imaging are recommended.^1,2^  In the case of clear grade 3 or grade 4 injury, do not delay pharmaceutical interventions (thrombolytics and/or prostacyclin analogue therapy).^1^

- Visual
  - The Cauchy visual grading scale is helpful in visually assessing the grade of injury
  - If injury is clearly a grade 3 or 4 treatment should proceed with pharmacologic interventions in appropriately screened patients
  - If grade 2 injury, treatment depends on center protocols
  - If unclear, then additional perfusion assessment should occur
- Doppler examination
  - Assessment of limb and digit pulses is performed transcutaneously to assess lack of perfusion
- Indocyanine green (ICG) Microangiography (SPY/LUNA)
  - ICG contrast is used to assess microvascular perfusion with ultraviolet light
  - Performed at bedside
- Angiography
  - Catheter directed contrasted fluoroscopy to assess microvascular perfusion
  - Used in patients who undergo IA thrombolytics per Interventional Radiology for diagnosis and for evaluation of response to treatment
- Nuclear Bone Scan (Tc-99)
  - 3 phase scan with delayed imaging and technetium contrast tracer to assess macrovascular, microvascular, soft tissue and bone perfusion
  - Early phase used to assess eligibility for pharmaceutical intervention

**III.** **Classification** – Assess when affected area is RE-WARMED. Visual assessment^3^  of ischemia is challenging, err on the side of the higher grade and re-evaluate on day 2. Injury may continue to demarcate within the first 72 hours.

- Superficial
  - Grade 1
    - Partial thickness skin freezing with numbness, erythema, hyperemia and the absence of blisters or cyanosis.
    - Healing likely with supportive cares.
- Superficial/Severe
  - Grade 2
    - Full thickness dermal freezing with erythema, hyperemia, and blistering.
      - Blisters may be serous and/or milky (formation of blisters is often delayed).
    - Cyanosis remains on the distal phalanx and does not cross a joint.
    - Amputation risk low in absence of infection or additional injury.
    - Amputation risk:^3^ 0-1% 95% CI (0,3).
- Severe
  - Grade 3 – Cauchy et al. 2001 combined intermediate and proximal phalanx involvement. This can be divided into 3a – involvement of the intermediate phalanx distal to the proximal interphalangeal joint and 3b – involving proximal phalanx or the proximal interphalangeal joint. Note – thumb and great toe only have distal and proximal phalanx, so cannot have 3a injury.
    - Dermal and subcutaneous freezing with hemorrhagic blisters and cyanosis. Skin necrosis and eschar formation after two weeks.
    - Amputation risk:^3^
      - 3a – 23-39% 95% CI (10,52)
      - 3b – 60-83% 95% CI (45,100)
  - Grade 4 – full thickness dermal, subcutaneous tissue, muscle, tendon and bone freezing with mottled deep red or cyanotic coloring involving the metacarpals and metatarsals with mummification of tissues after several weeks.
    - Amputation risk:^3^  98-100% 95% CI (93,100)
- Each digit is graded independently and treatment is based on the most severe grade. The Hennepin Frostbite Score may be used to assess the impact of interventions for each patient. ^4,5^ Either formal imaging or visual assessment may be used for the ‘at risk’ assessment with the score to track amputation outcomes and salvage rate.

**IV.** Interventions – general rule: urgency for treatment increases as grade increases. From the time of rewarming, each hour in delay of pharmaceutical intervention results in decreases in limb salvage.

Evaluate eligibility for prostacyclins and/or thrombolysis based on clinical grading and imaging results.

Thrombolytics: Alteplase and tenecteplase have reported use in the U.S. and Canada.

- Post thrombolysis anticoagulation is required and varies widely by center (see Supplementary Table 9).

Prostacyclins: iloprost has FDA approval for use in frostbite. Centers have used epoprostenol (few reports, clinical trial).

- Grade 1 or 2 AND adequate perfusion on perfusion imaging
  - Proceed with supportive care
- Grade 2, 3, or 4 OR impaired perfusions on perfusion imaging
  - Consider thrombolytics within 24 hours of rewarming
  - Consider prostacyclin analogue within 72 hour of rewarming
    - Note: Thrombolysis and prostacyclin analogues may be given in conjunction when presentation within 24 hours of rewarming.

Supportive cares:

- Local Wound Care
  - Protective dressings with several layers of gauze to avoid trauma to the affected tissues
  - Topical antimicrobials depending on depth of injury
- Blister management varies, but typically leave hemorrhagic blisters intact for 48-72 hours. Serous blisters may be drained, debrided, or left intact if not limiting range of movement.
- Edema Management
  - Elevate affected extremities
  - Light compression
- Pain control
- Tetanus prophylaxis
- Anti-platelet agents: Aspirin or Ibuprofen
- Rehabilitation

**V.** Surgical management: Not typically protocolized. Some general guidance based on authors expertise:

- Delayed, planned readmission to allow for demarcation of tissue and preserve limb length.
  - May use advanced imaging to identify viability of limbs/digits for early surgical management on day 8 or later
- Early Debridement: if there is concern for infection
- Amputation: mummified or clearly demarcated digits/extremities

Reconstruction: skin grafts or flaps for full thickness tissue loss and preserve length

1. Wibbenmeyer, L. *et al.* American Burn Association Clinical Practice Guidelines on the Treatment of Severe Frostbite. *J. Burn Care Res.* **45**, 541–556 (2023).

2. McIntosh, S. E. *et al.* Wilderness Medical Society Clinical Practice Guidelines for the Prevention and Treatment of Frostbite: 2024 Update. *Wilderness Environ. Med.* **35**, 183–197 (2023).

3. Cauchy, E., Chetaille, E., Marchand, V. & Marsigny, B. Retrospective study of 70 cases of severe frostbite lesions: a proposed new classification scheme. *Wilderness Environ. Med.* **12**, 248–255 (2001).

4. Nygaard, R. M., Whitley, A. B., Fey, R. M. & Wagner, A. L. The Hennepin Score. *J. Burn Care Res.* **37**, e317–e322 (2016).

5. Nygaard, R. Hennepin Frostbite Score Worksheet. <https://www.rachelnygaard.com/frostbite>.
